# Supplementary figures and images for: Disordered proteins interact with the chemical environment to tune their protective function during drying
Source: eLife. 2024 Nov 19;13:RP97231. doi: 10.7554/eLife.97231 (PMC11575898; doi:10.7554/eLife.97231)

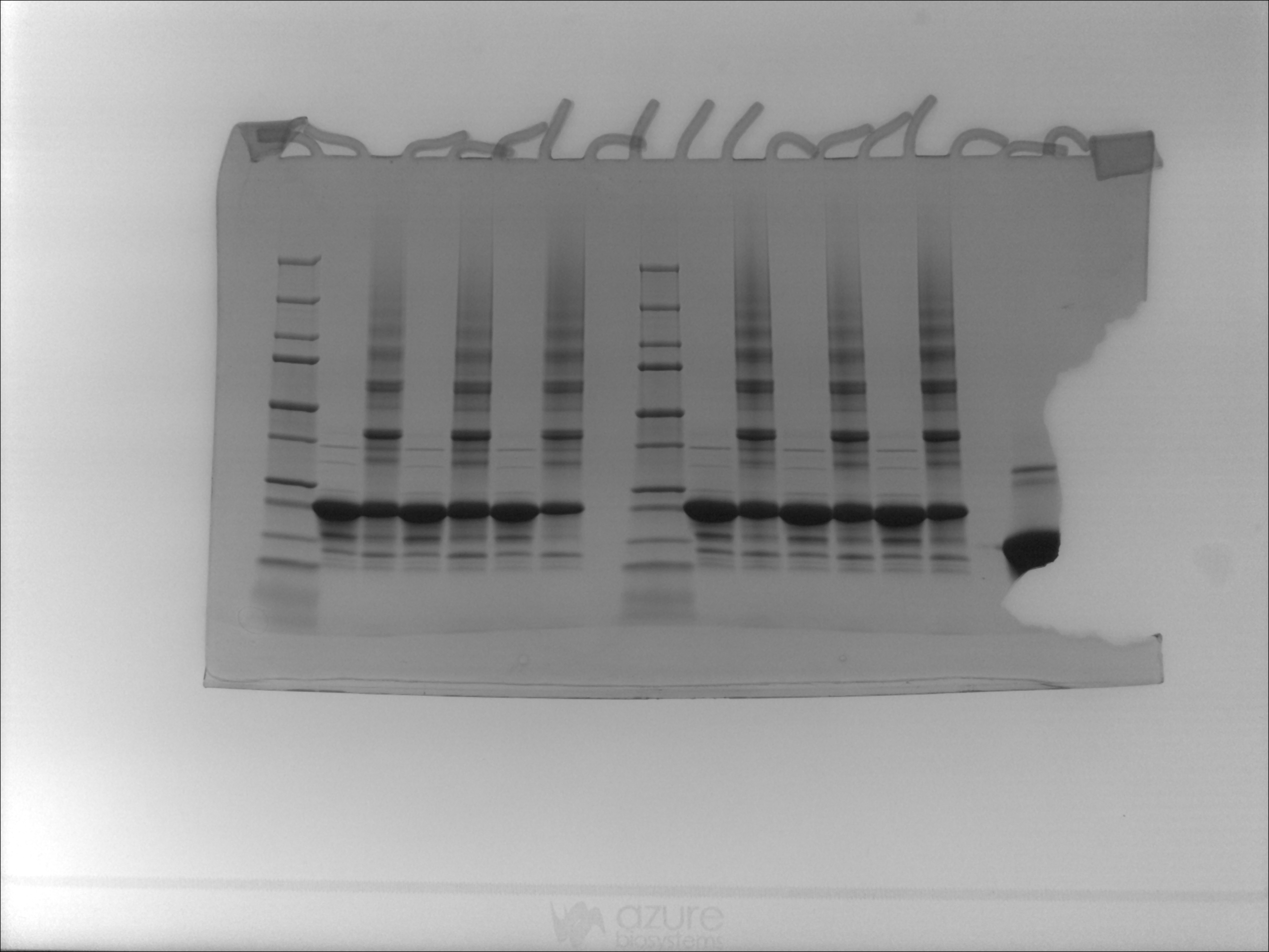

Supplement: Figure 4—figure supplement 2—source data 1. [file elife-97231-fig4-figsupp2-data1.zip › Raw gel files/AtLEA3-3_150uM-200uM.tiff]

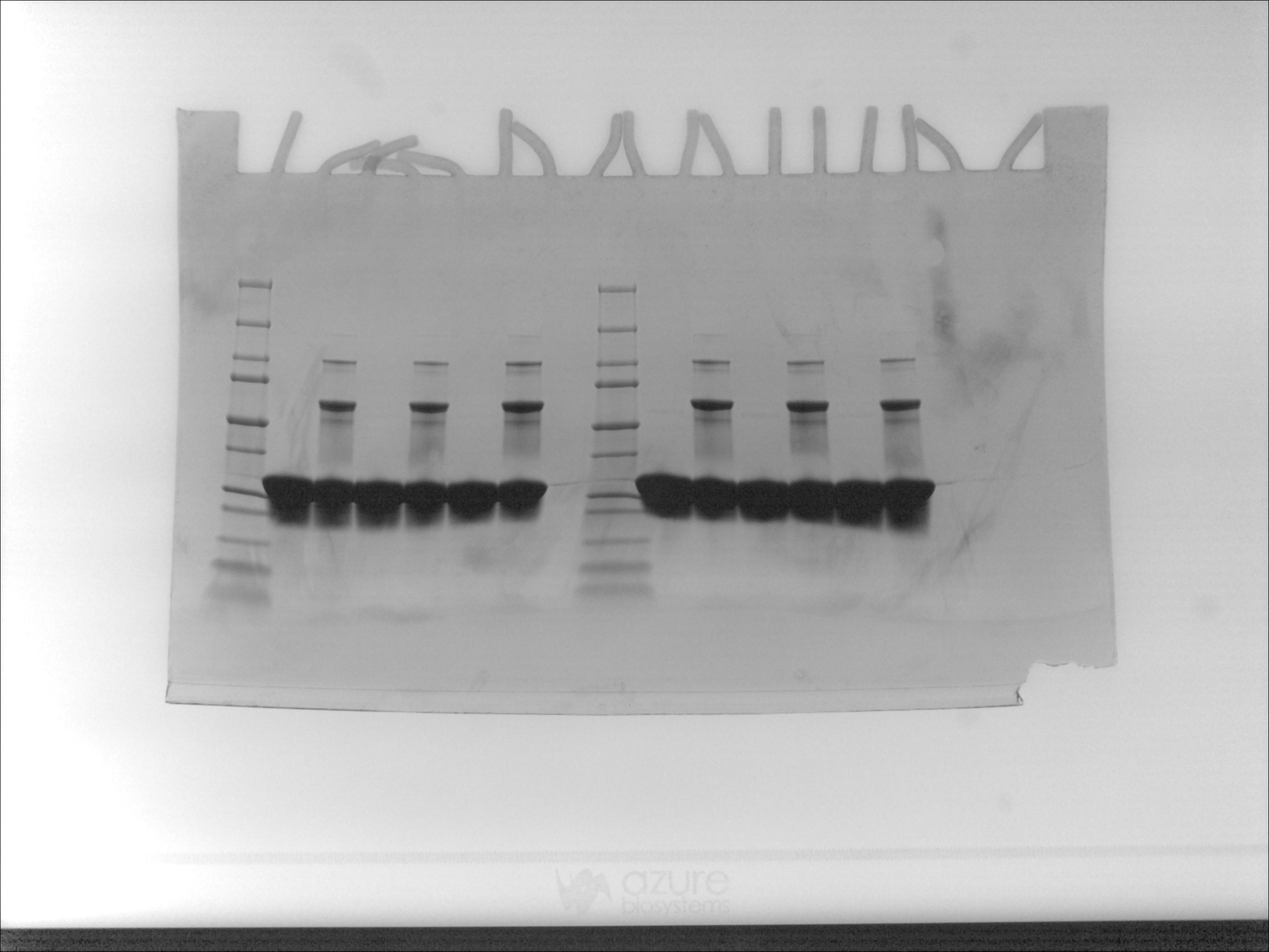

Supplement: Figure 4—figure supplement 2—source data 1. [file elife-97231-fig4-figsupp2-data1.zip › Raw gel files/HeLEA68614_150uM-200uM.tiff]

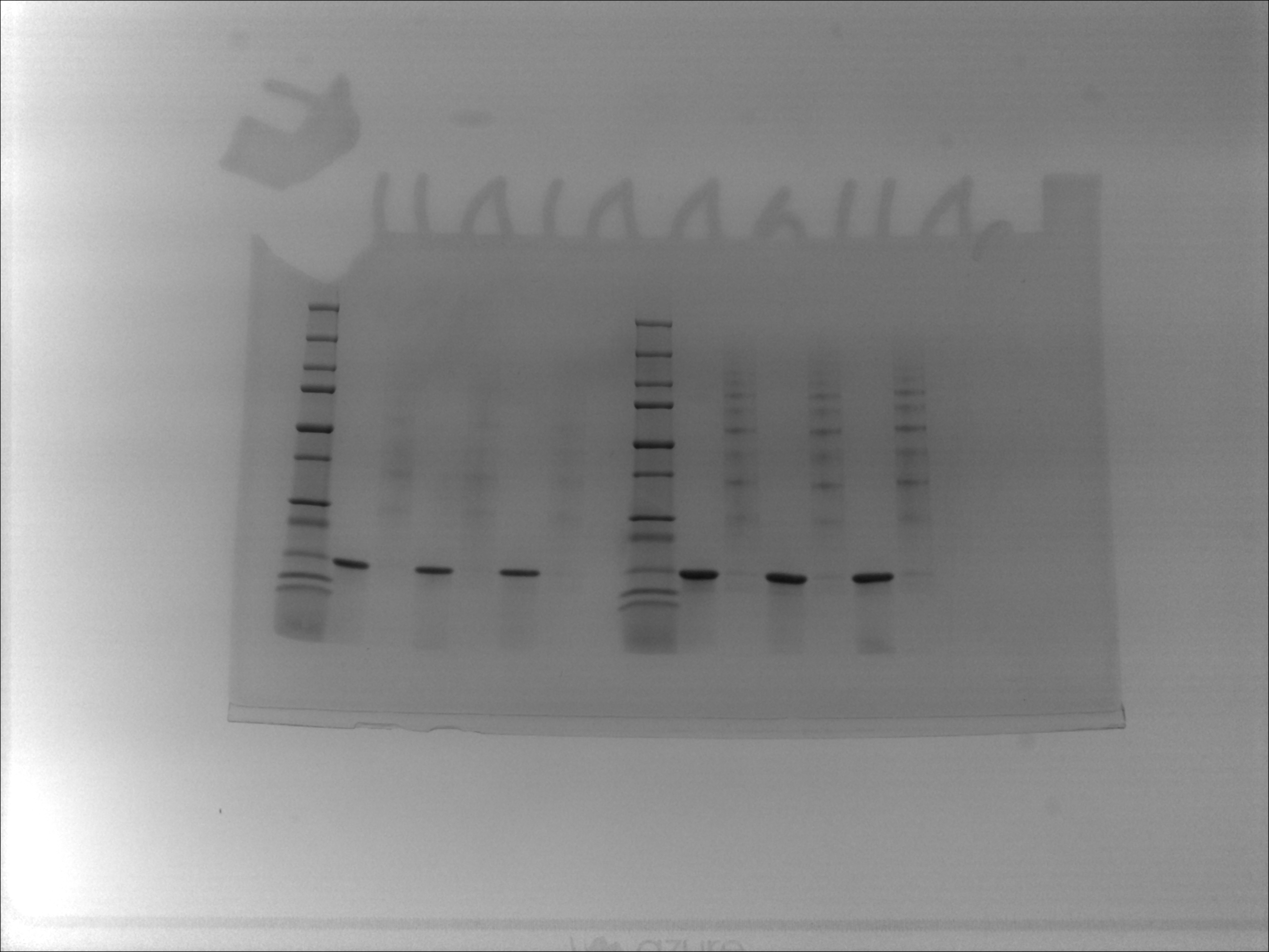

Supplement: Figure 4—figure supplement 2—source data 1. [file elife-97231-fig4-figsupp2-data1.zip › Raw gel files/AtLEA4-2_5uM-50uM.tiff]

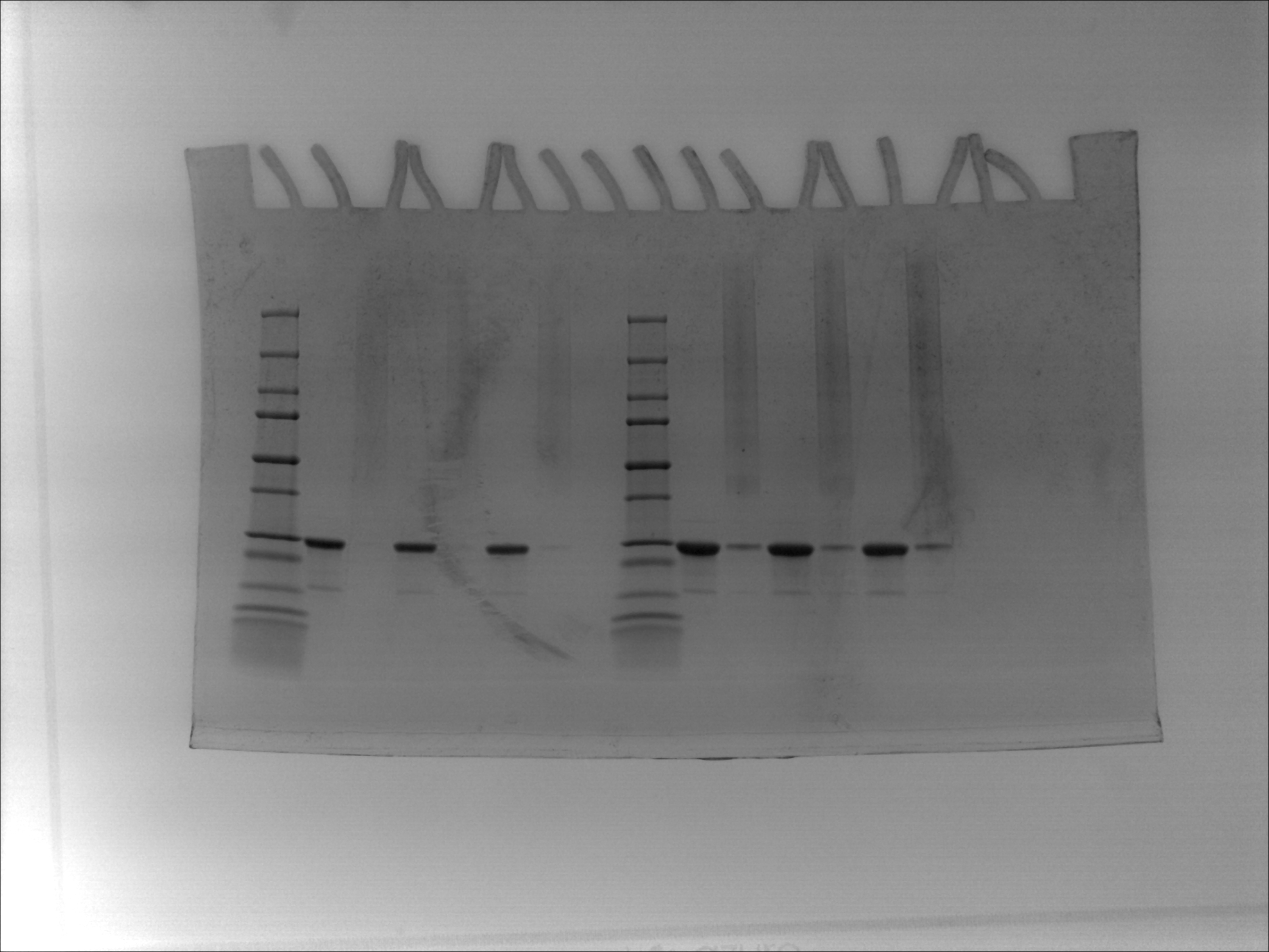

Supplement: Figure 4—figure supplement 2—source data 1. [file elife-97231-fig4-figsupp2-data1.zip › Raw gel files/AvLEA1C_25uM-50uM.tiff]

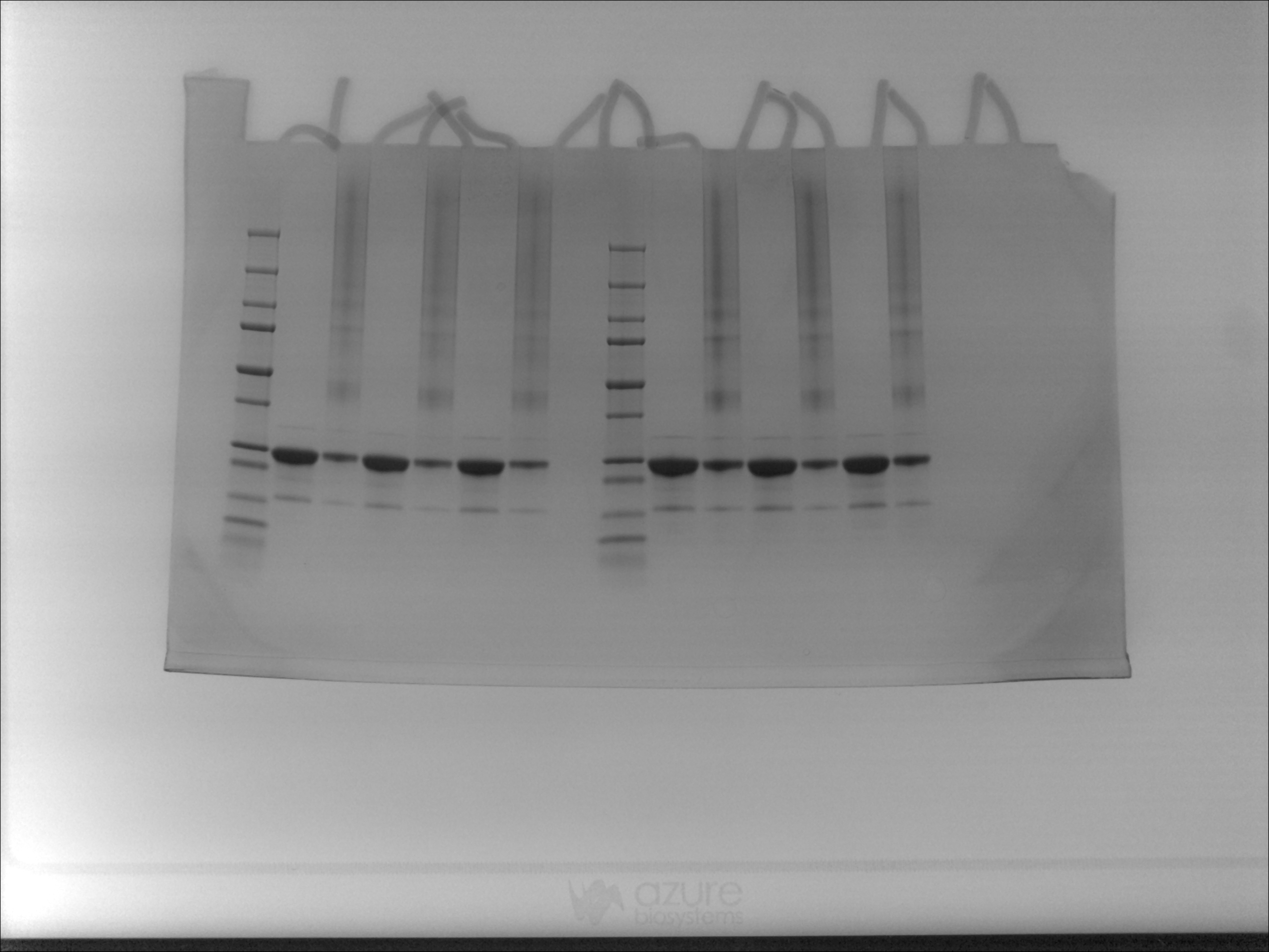

Supplement: Figure 4—figure supplement 2—source data 1. [file elife-97231-fig4-figsupp2-data1.zip › Raw gel files/AvLEA1C_75uM-100uM.tiff]

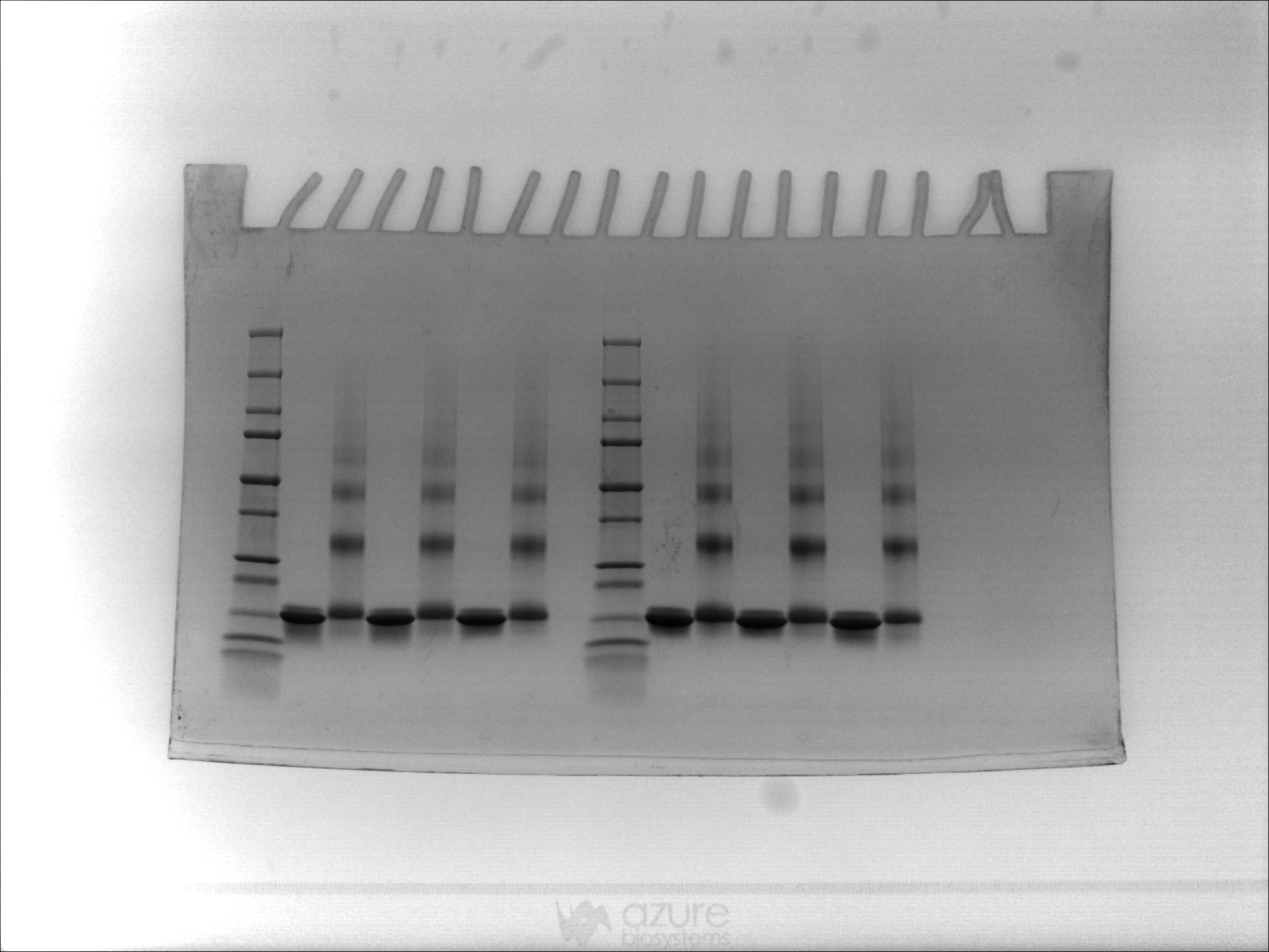

Supplement: Figure 4—figure supplement 2—source data 1. [file elife-97231-fig4-figsupp2-data1.zip › Raw gel files/AavLEA1_ 75uM-100uM.tiff]

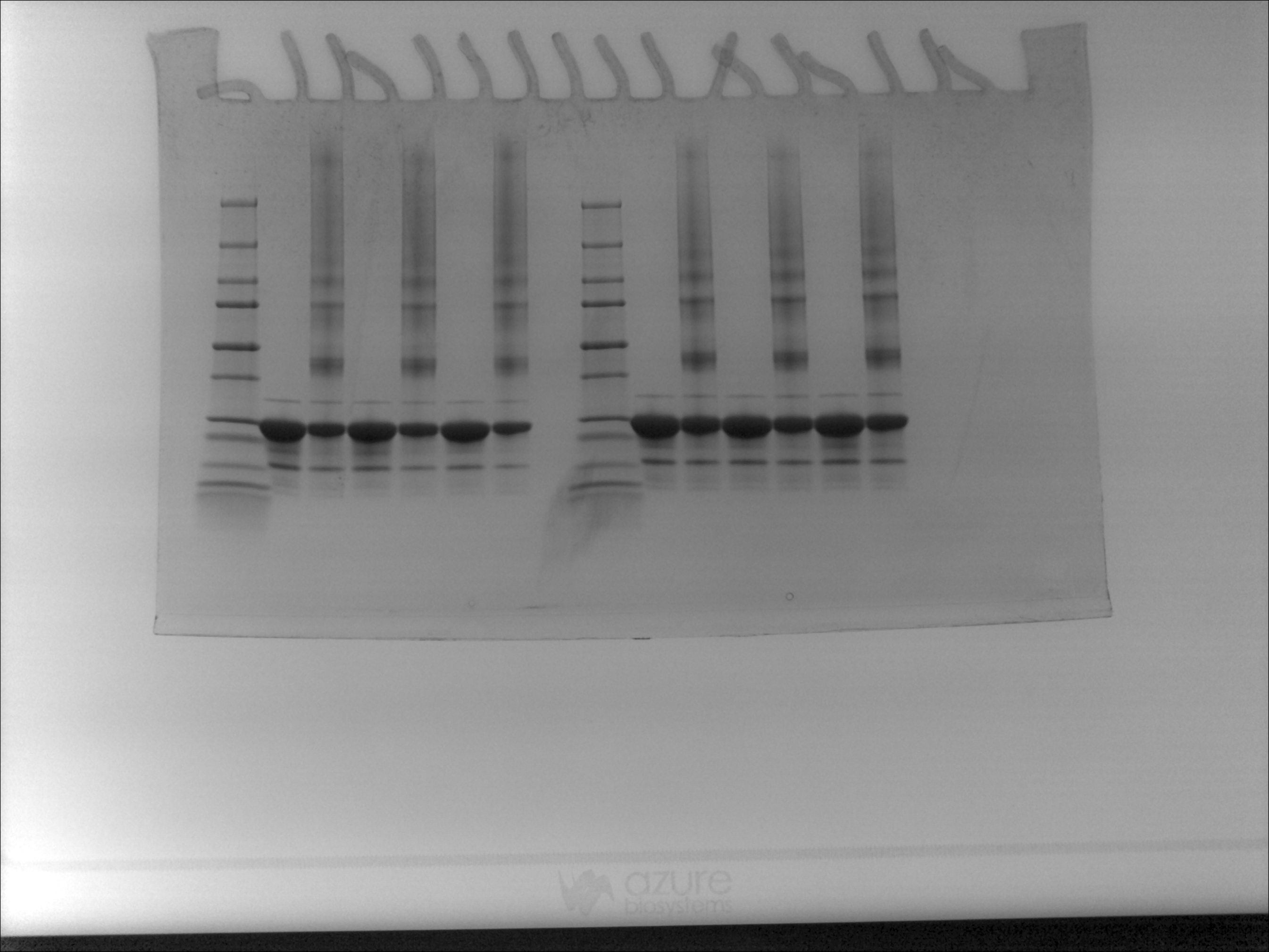

Supplement: Figure 4—figure supplement 2—source data 1. [file elife-97231-fig4-figsupp2-data1.zip › Raw gel files/AvLEA1C_150uM-200uM.tiff]

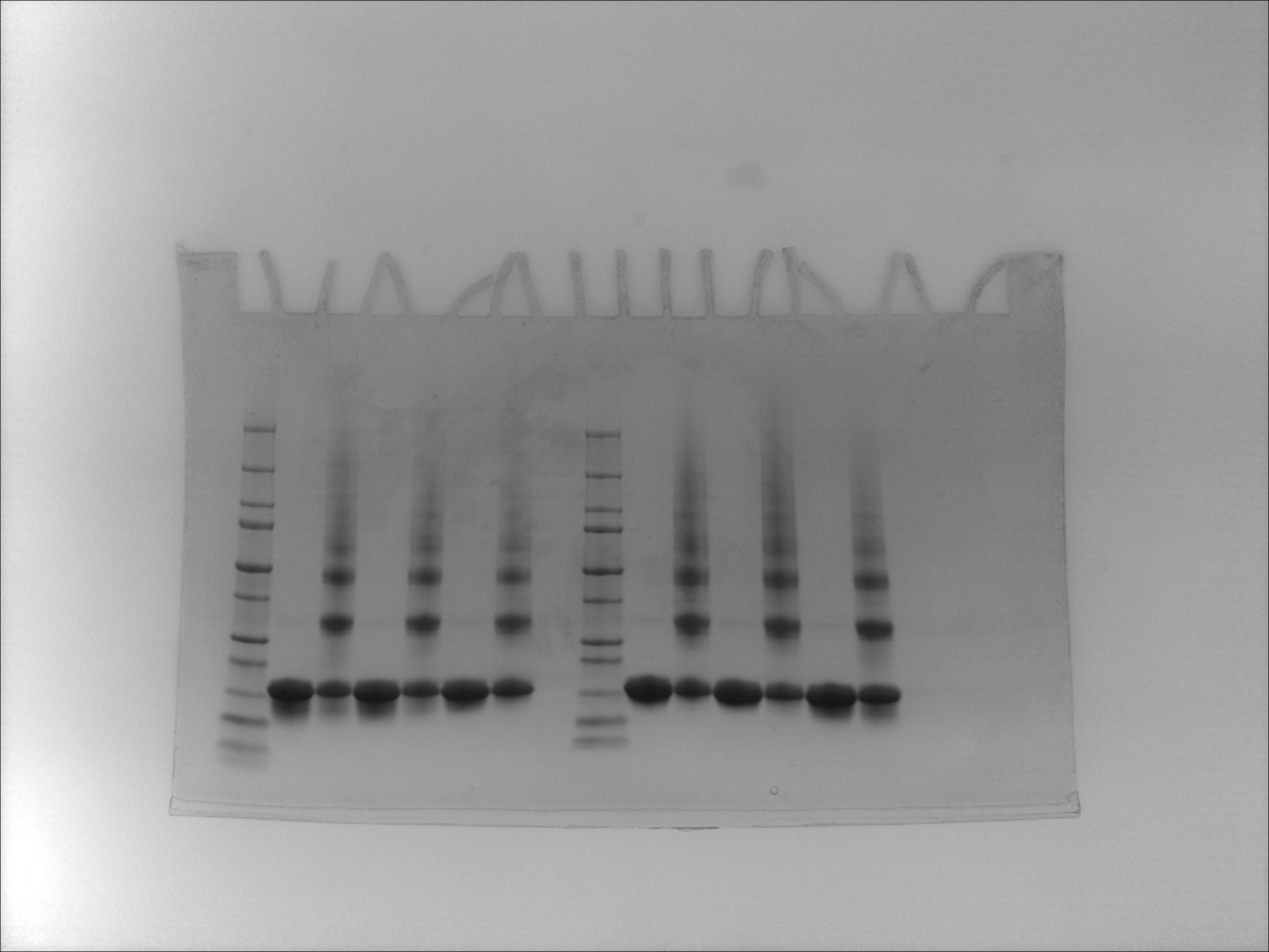

Supplement: Figure 4—figure supplement 2—source data 1. [file elife-97231-fig4-figsupp2-data1.zip › Raw gel files/AavLEA1_ 150uM-200uM.tiff]

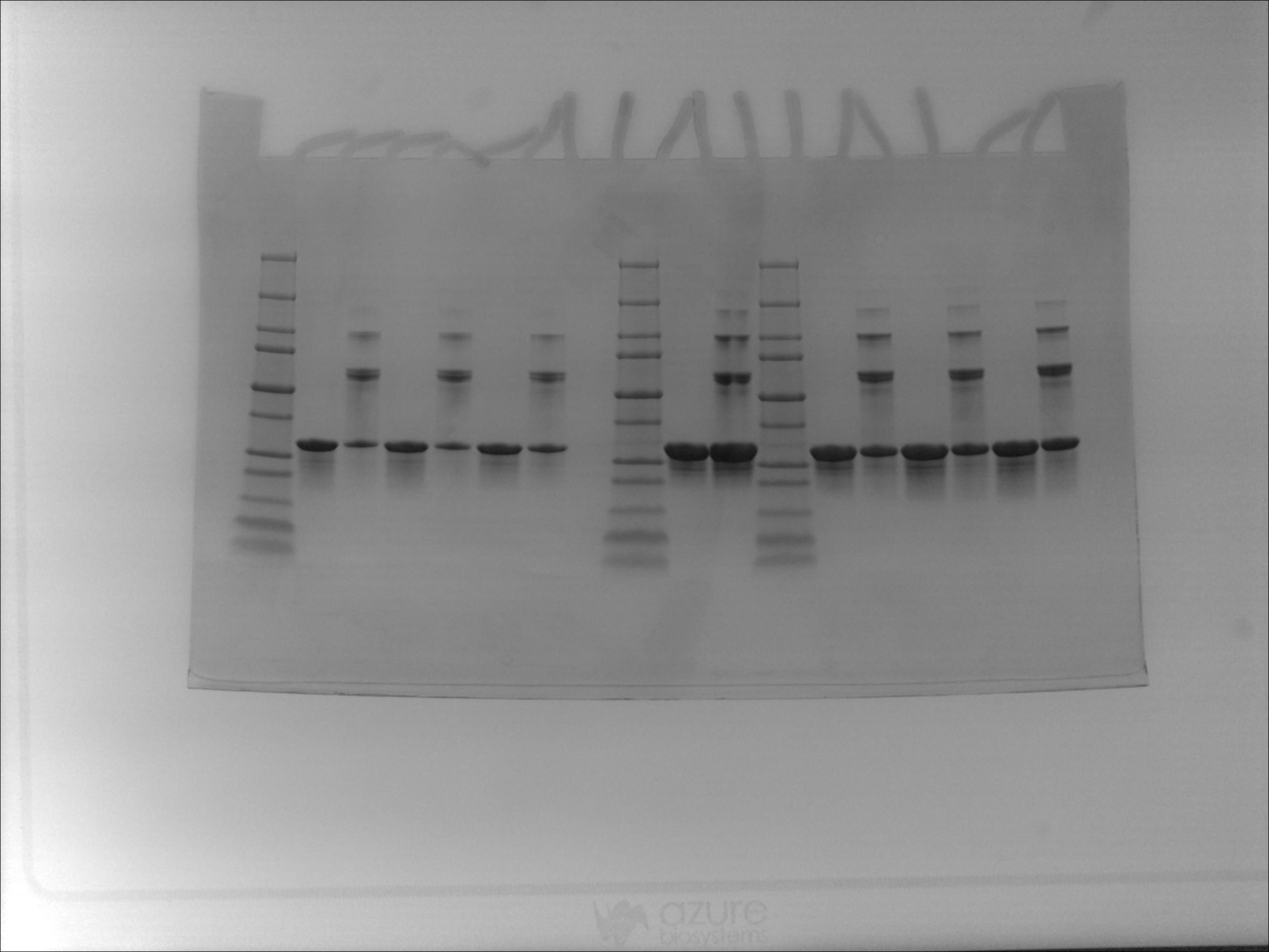

Supplement: Figure 4—figure supplement 2—source data 1. [file elife-97231-fig4-figsupp2-data1.zip › Raw gel files/HeLEA68614_25uM-50uM.tiff]

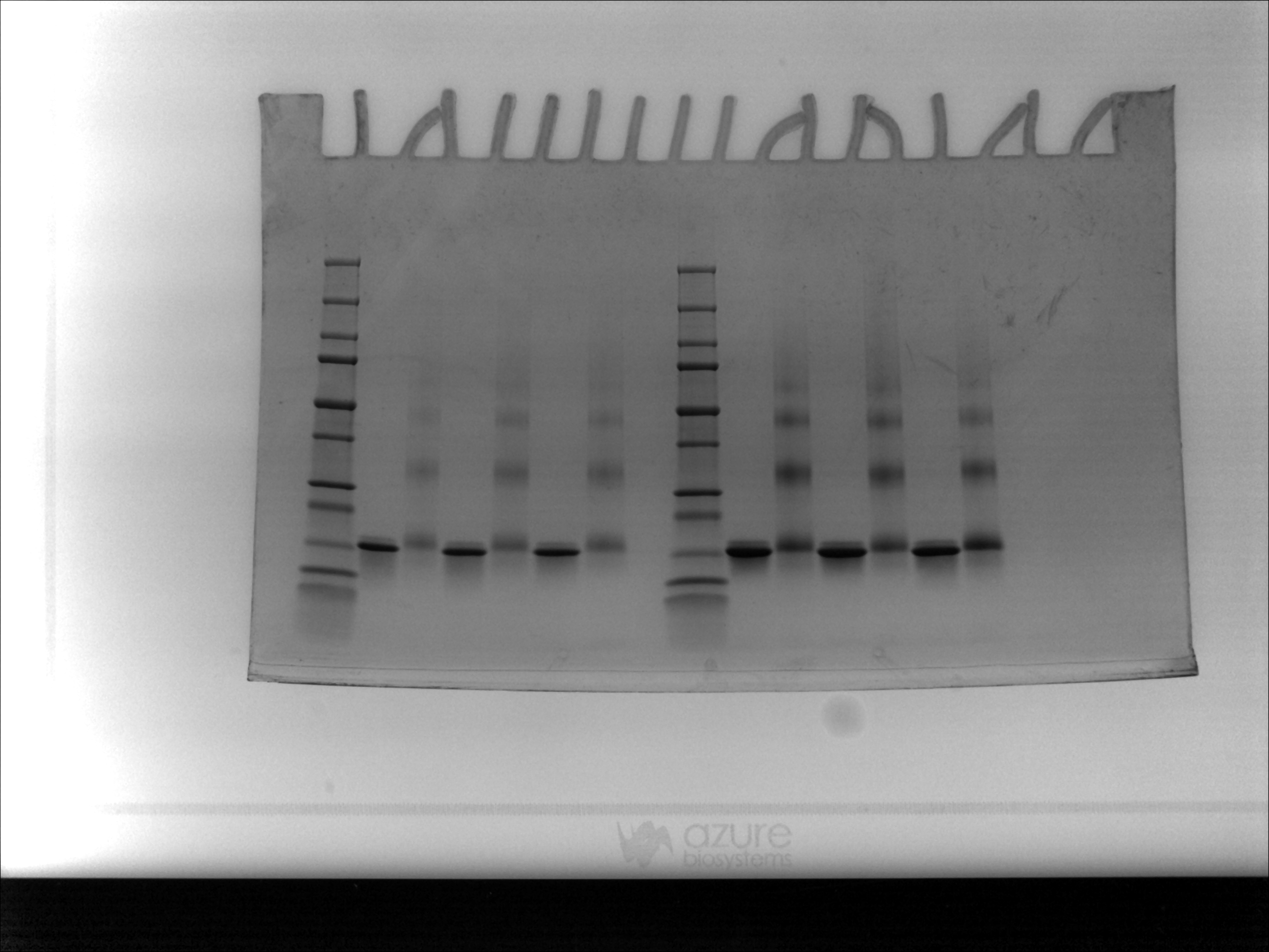

Supplement: Figure 4—figure supplement 2—source data 1. [file elife-97231-fig4-figsupp2-data1.zip › Raw gel files/AavLEA1_25uM-50uM.tiff]

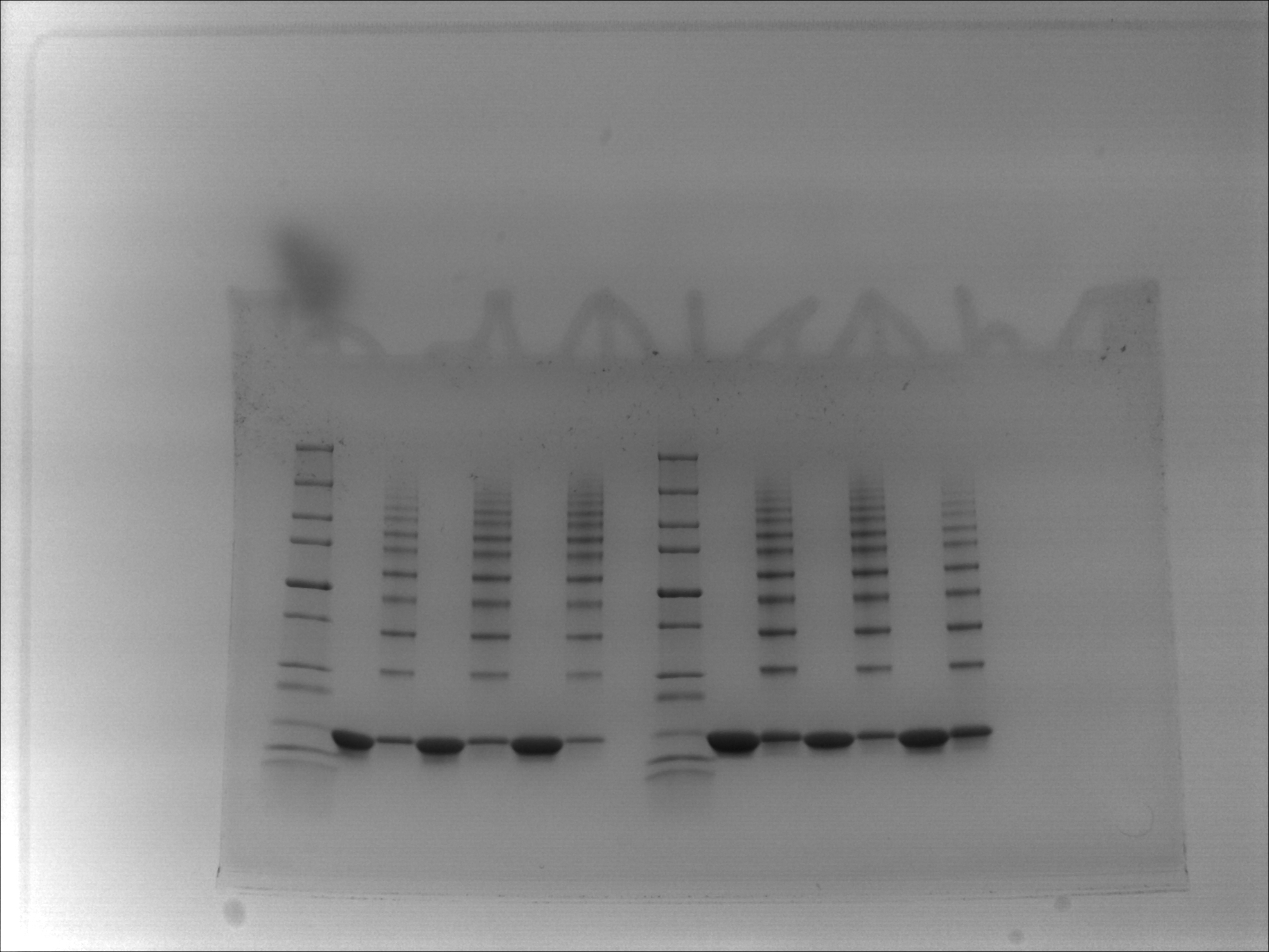

Supplement: Figure 4—figure supplement 2—source data 1. [file elife-97231-fig4-figsupp2-data1.zip › Raw gel files/AtLEA4-2_150uM-200uM.tiff]

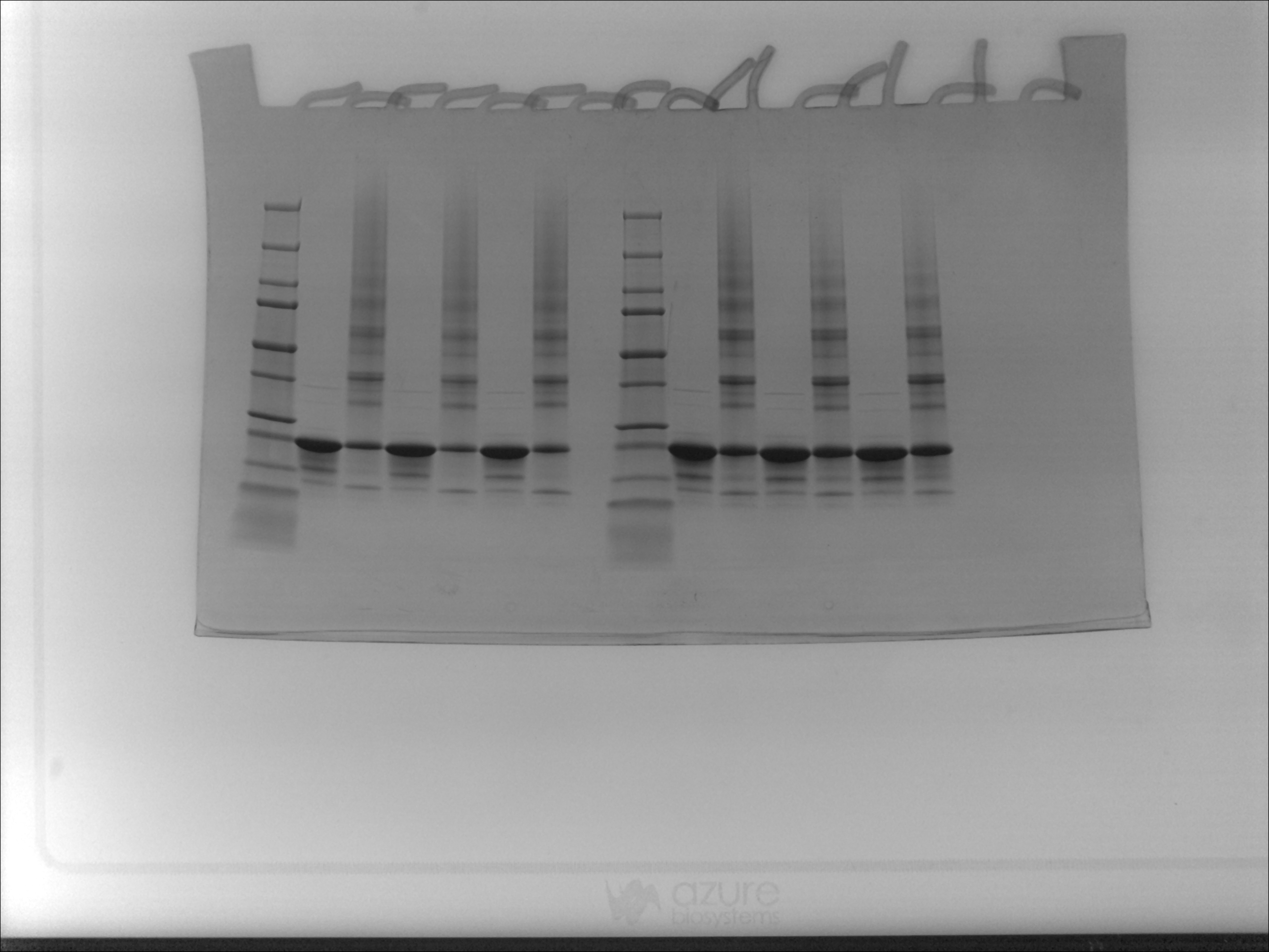

Supplement: Figure 4—figure supplement 2—source data 1. [file elife-97231-fig4-figsupp2-data1.zip › Raw gel files/AtLEA3-3_75uM-100uM.tiff]

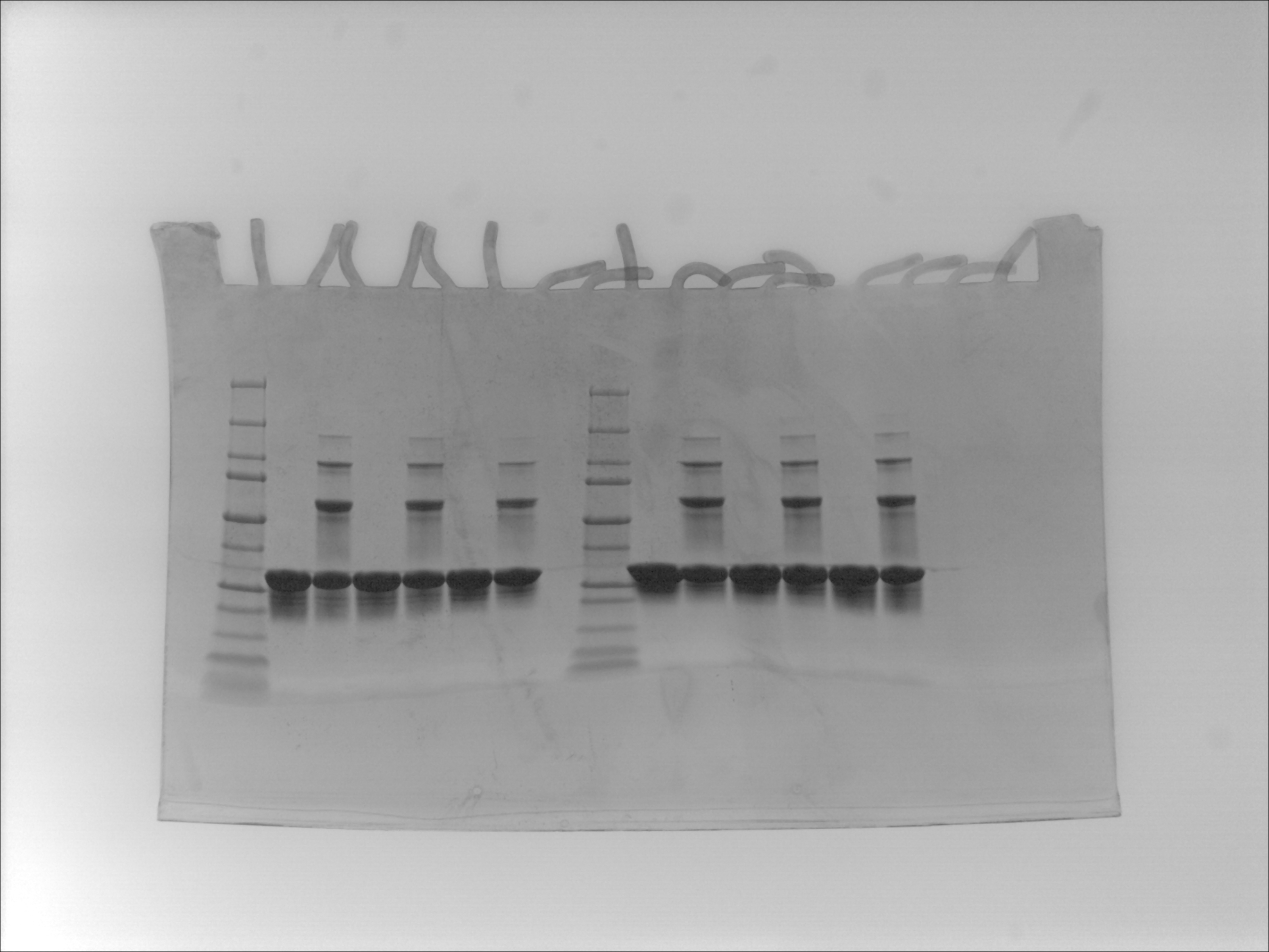

Supplement: Figure 4—figure supplement 2—source data 1. [file elife-97231-fig4-figsupp2-data1.zip › Raw gel files/HeLEA68614_75uM-100uM.tiff]

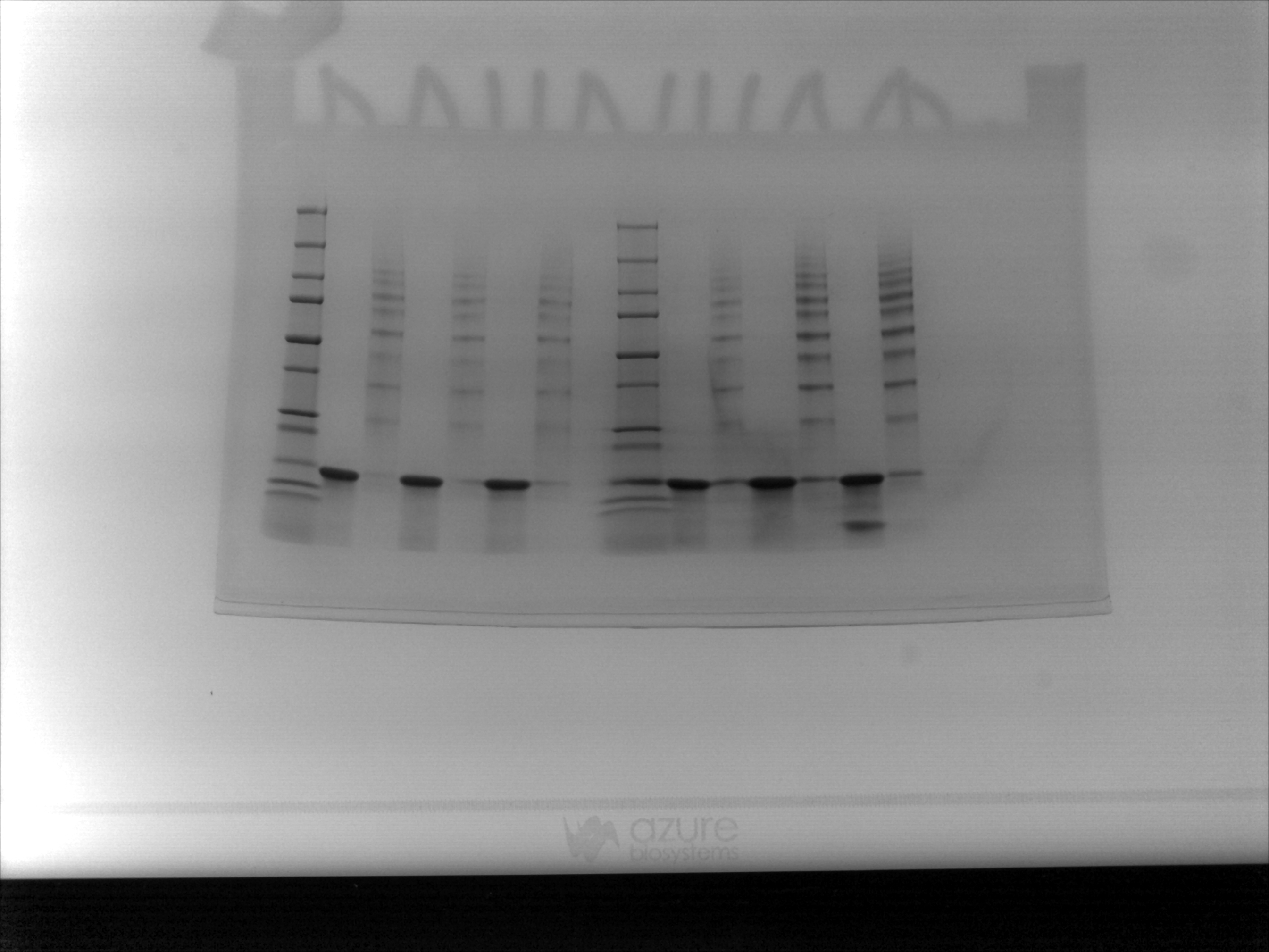

Supplement: Figure 4—figure supplement 2—source data 1. [file elife-97231-fig4-figsupp2-data1.zip › Raw gel files/AtLEA4-2_75uM-100uM.tiff]

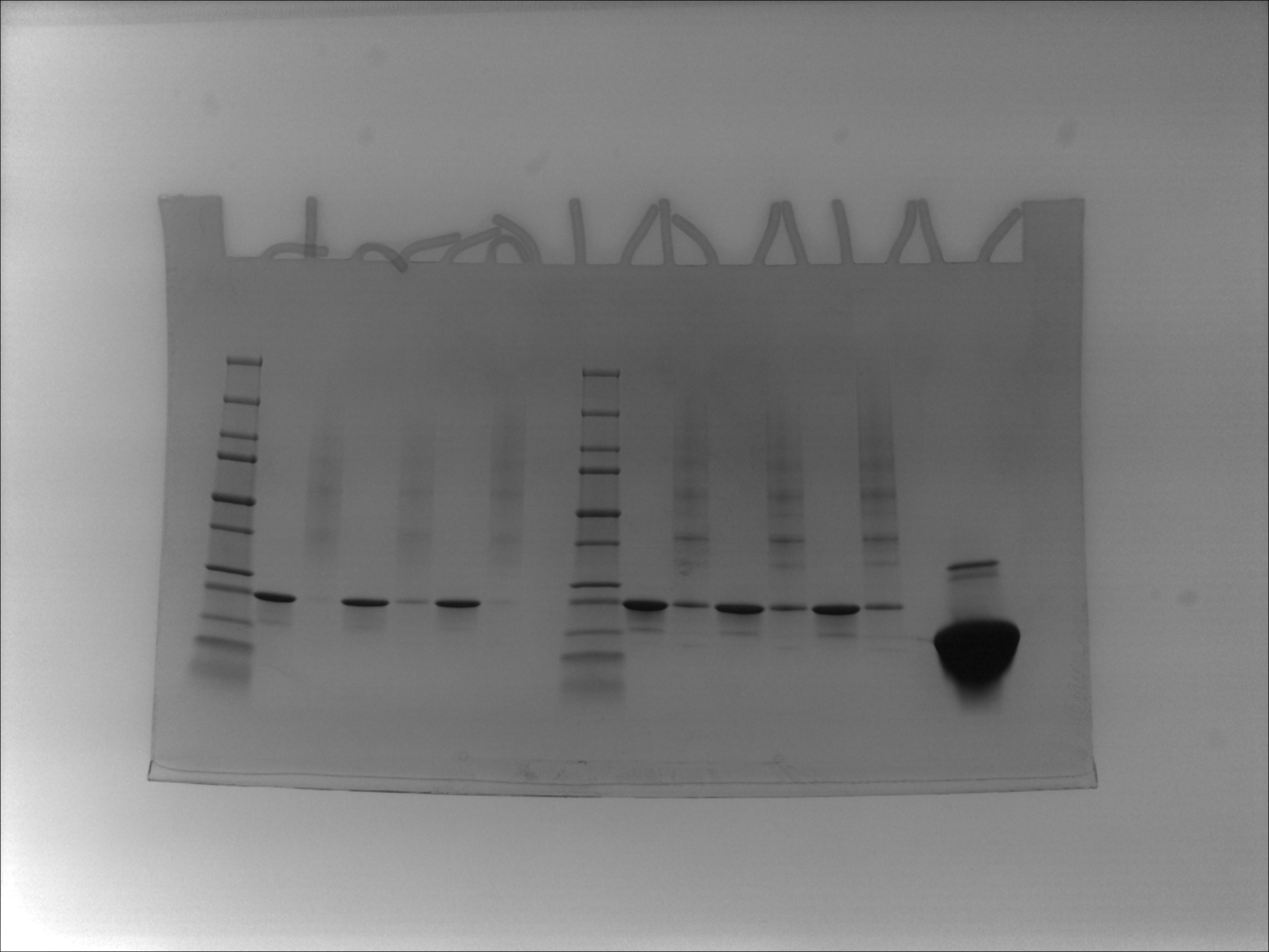

Supplement: Figure 4—figure supplement 2—source data 1. [file elife-97231-fig4-figsupp2-data1.zip › Raw gel files/AtLEA3-3_25uM-50uM.tiff]

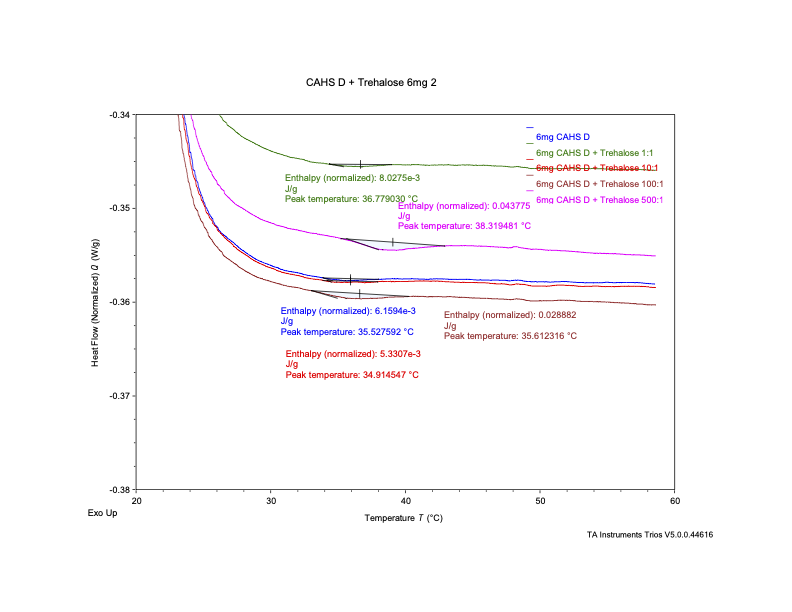

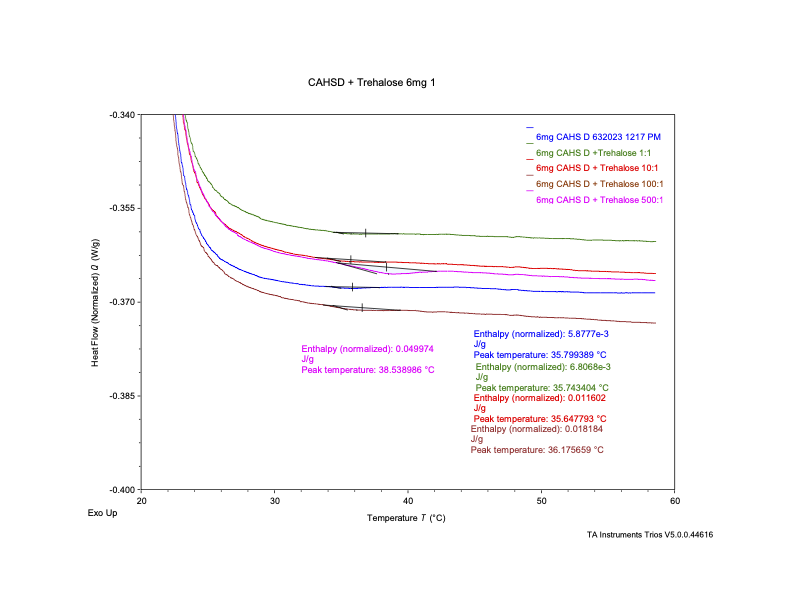


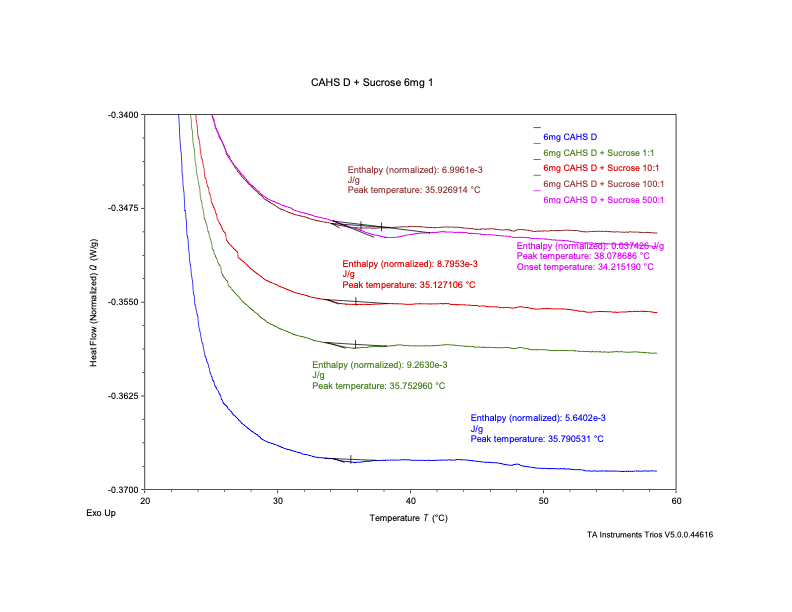

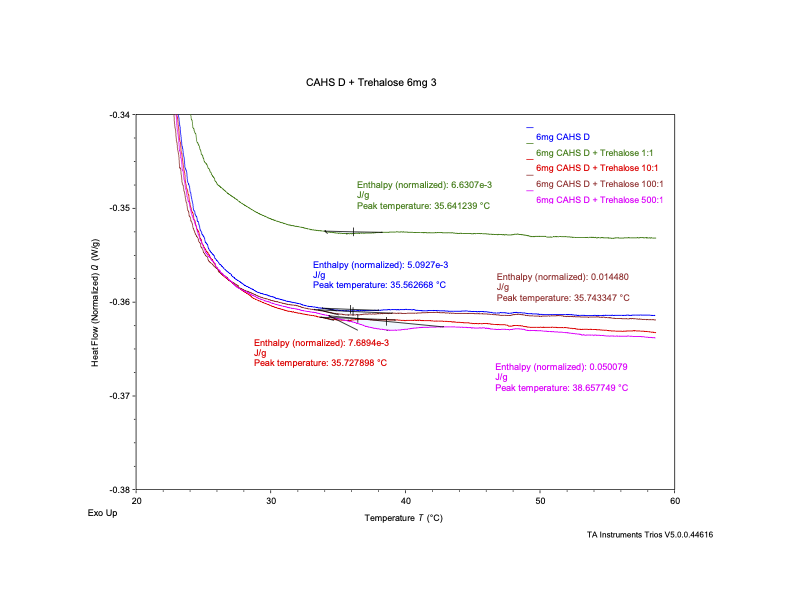

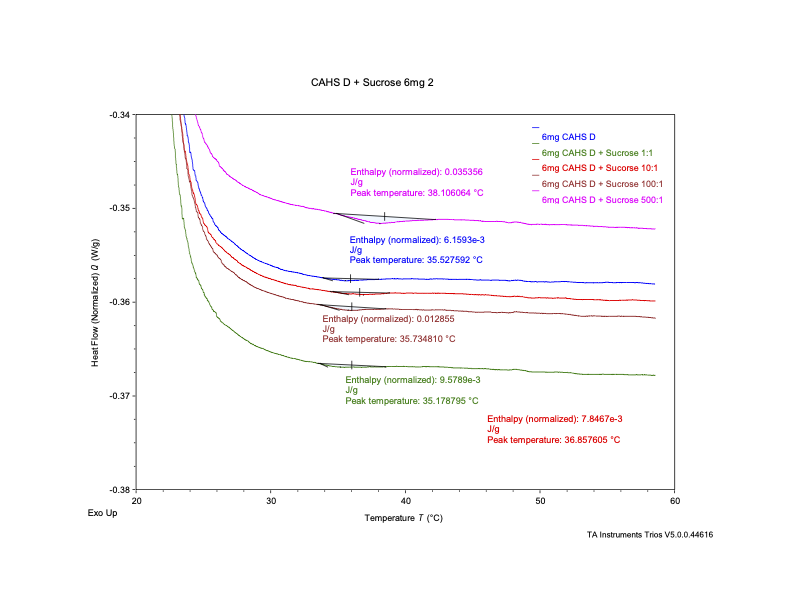

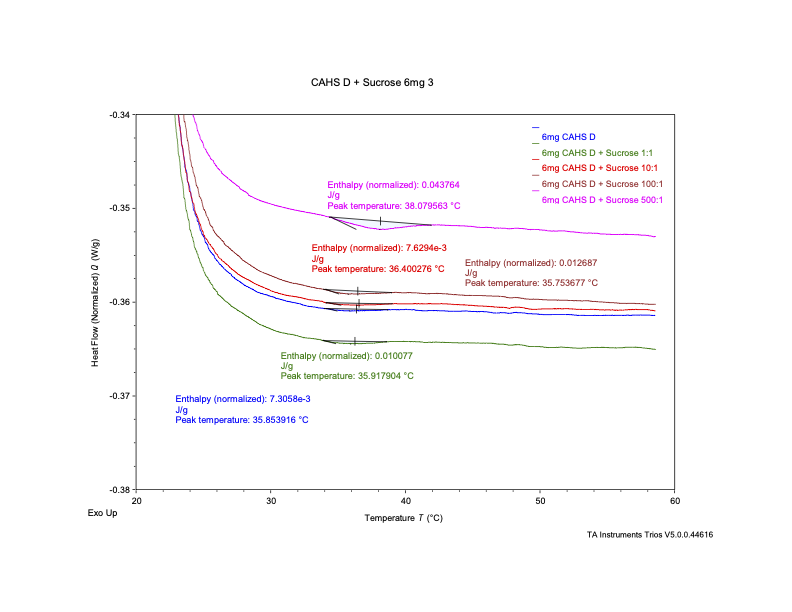

Supplement: Figure 5—source data 2. [file elife-97231-fig5-data2.docx]

Synergy Paper TFE equations

DSC thermograms for Betaine-CAHS D


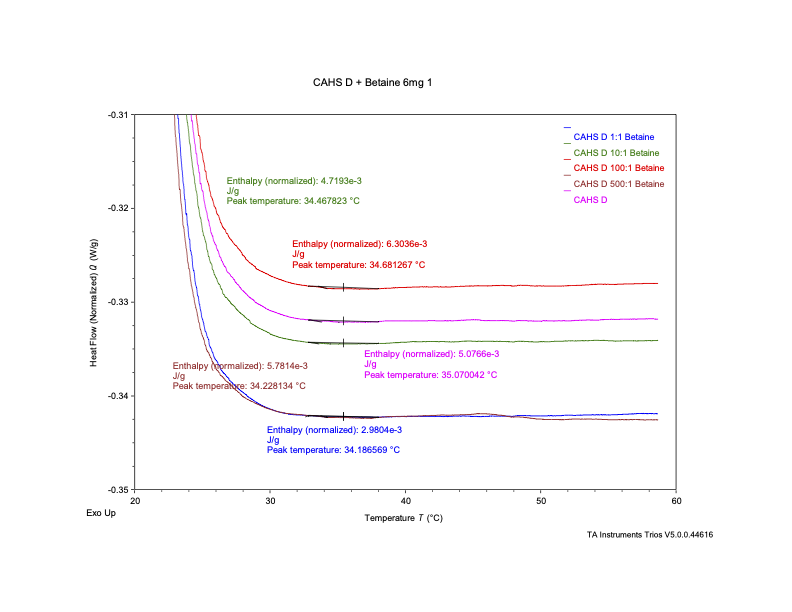


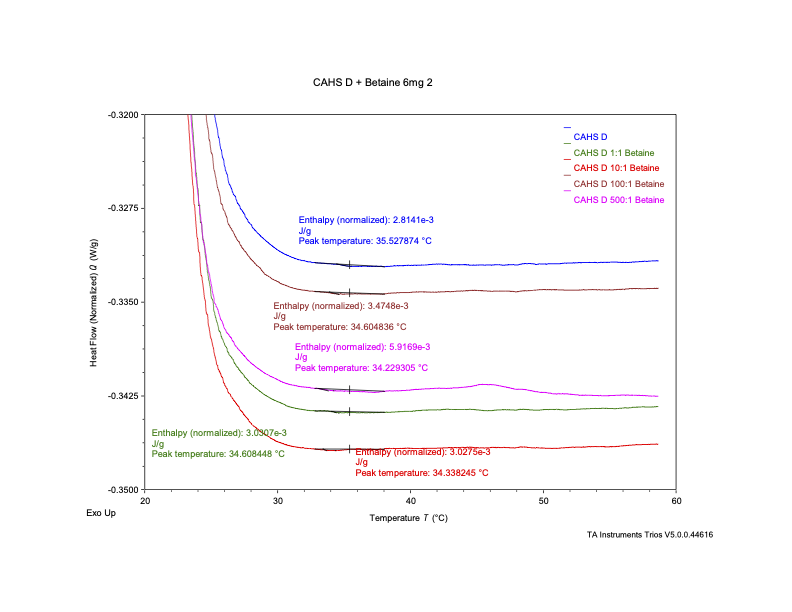

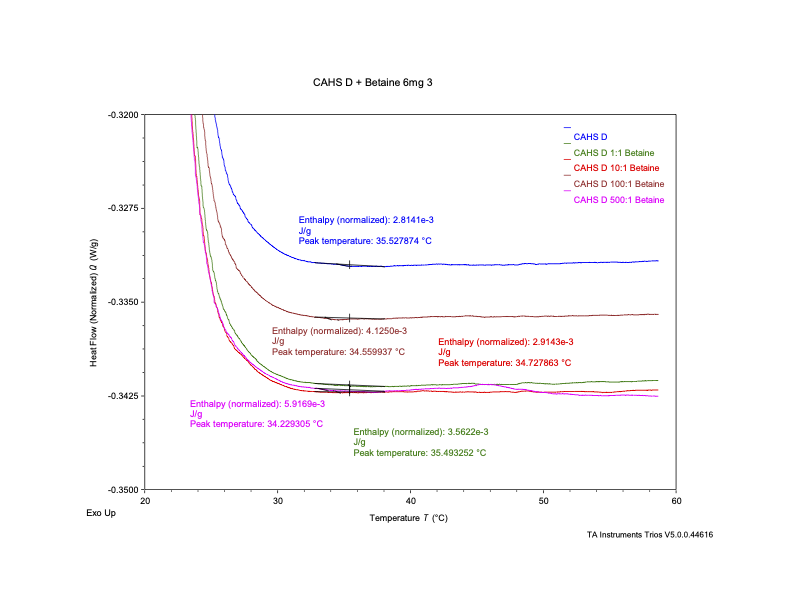


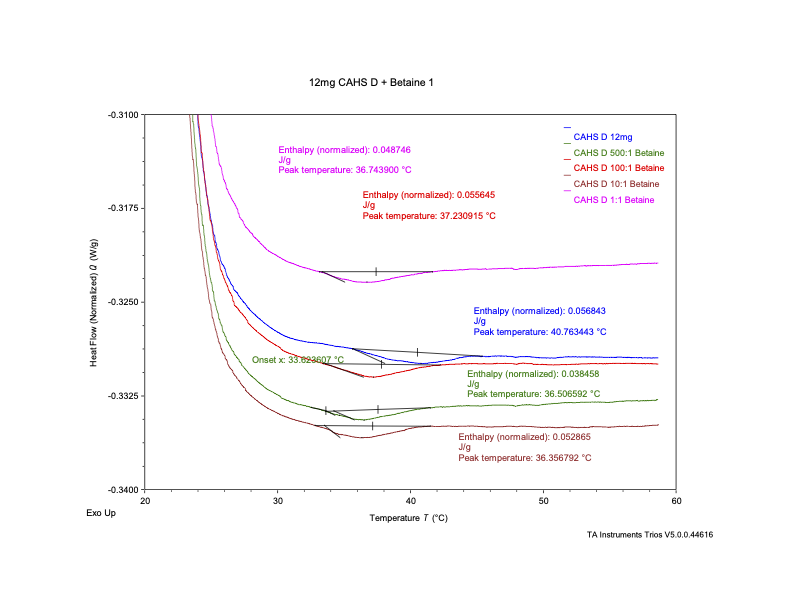


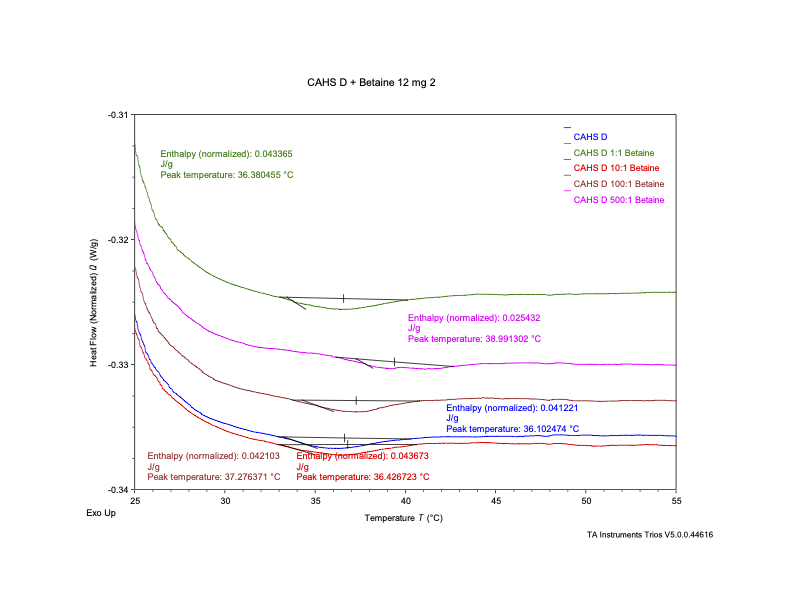


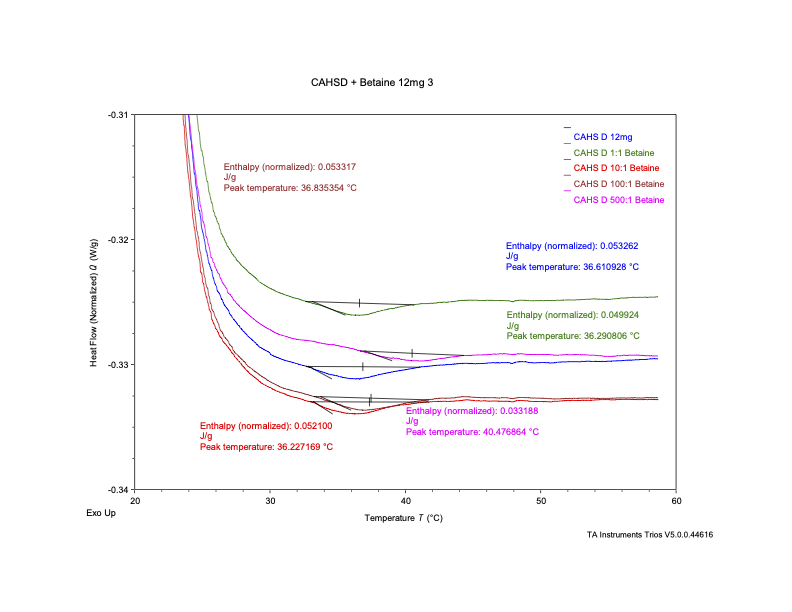

Supplement: Figure 6—source data 2. [file elife-97231-fig6-data2.docx]
