# Supplementary material for: Disordered proteins interact with the chemical environment to tune their protective function during drying
Source: eLife. 2024 Nov 19;13:RP97231. doi: 10.7554/eLife.97231 (PMC11575898; doi:10.7554/eLife.97231)

**AavLEA1**

1. 25 µM B) 50 µM

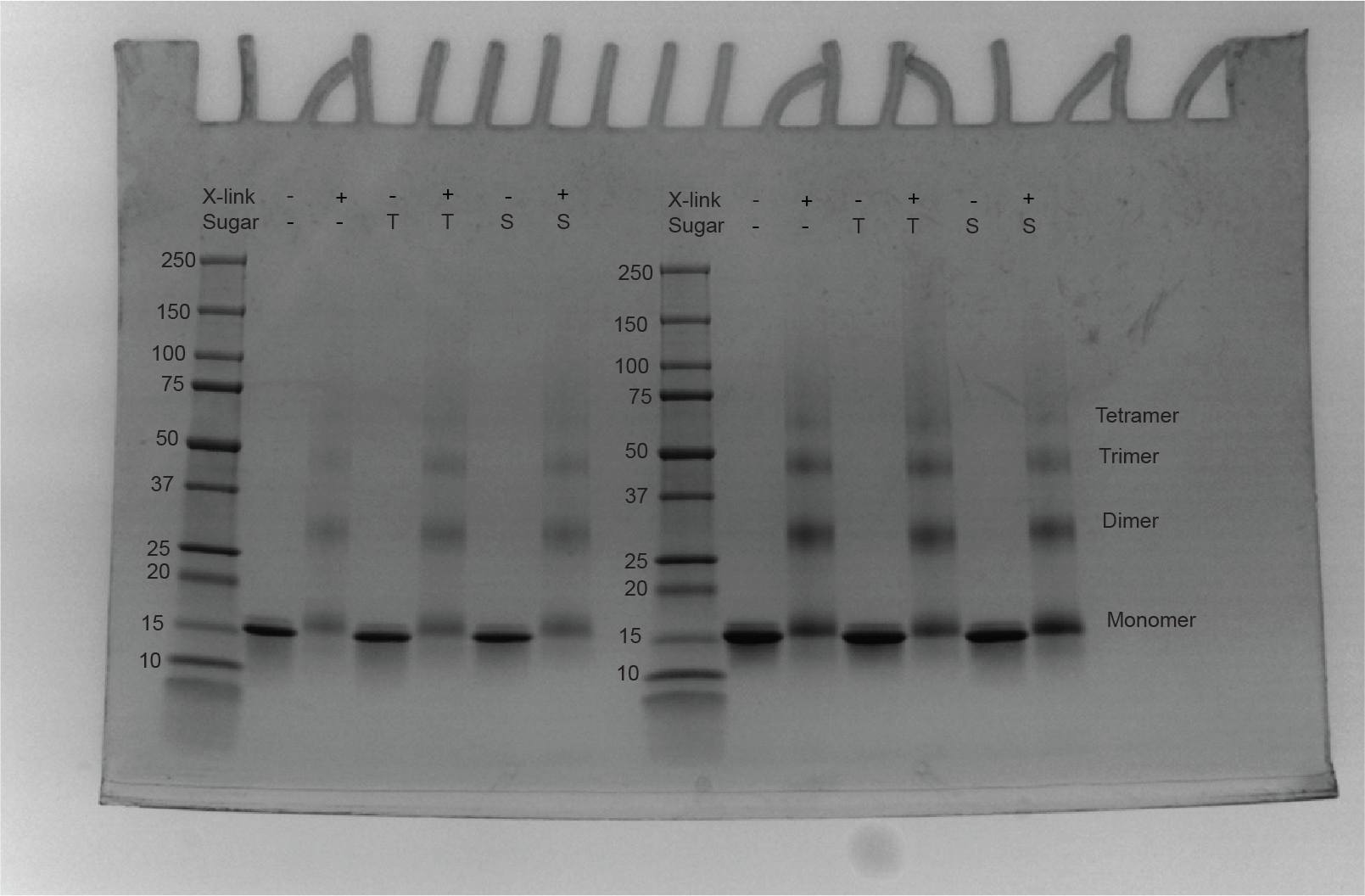


1. 75 µM D) 100 µM

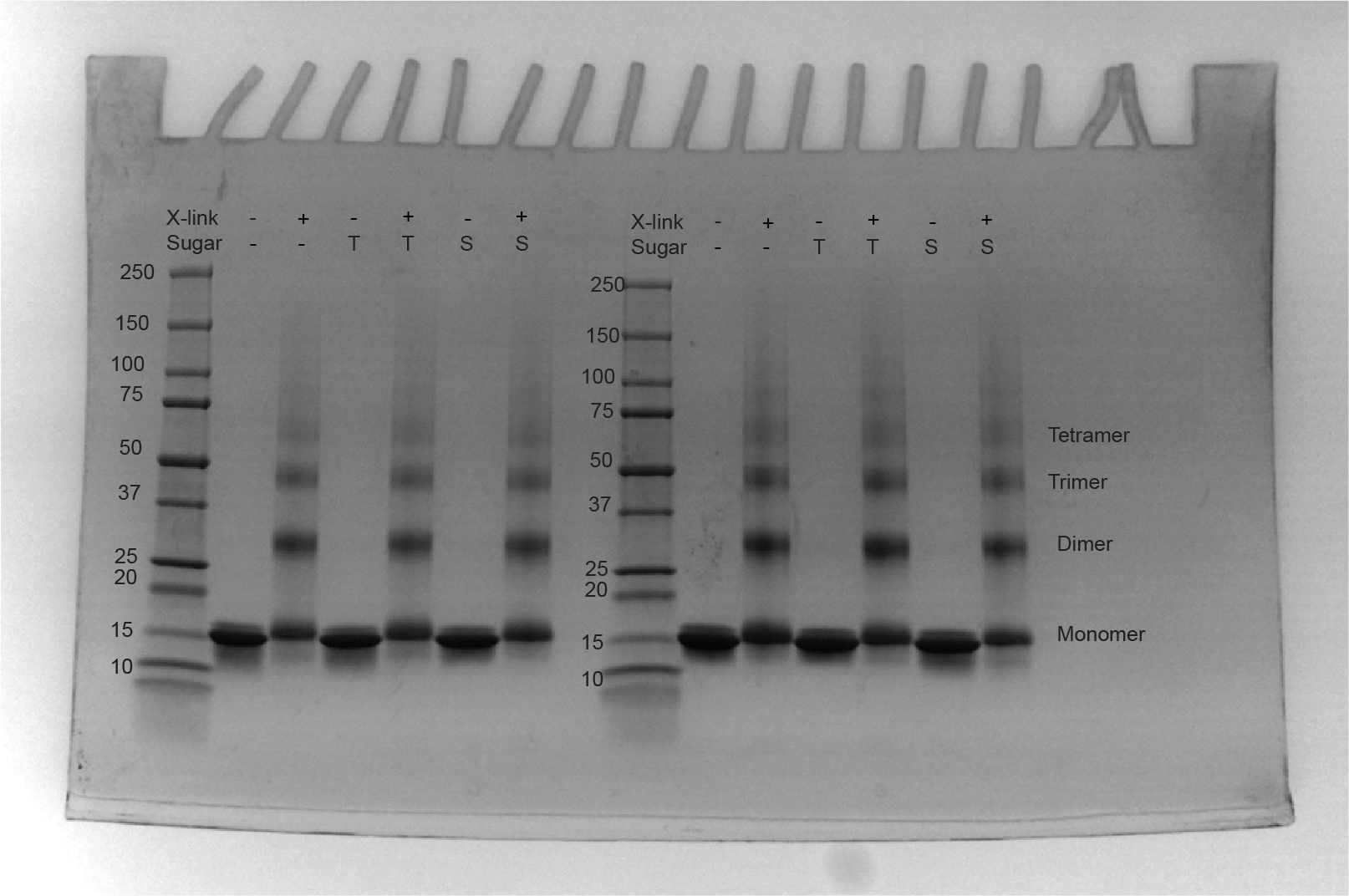


1. 150 µM F) 200 µM

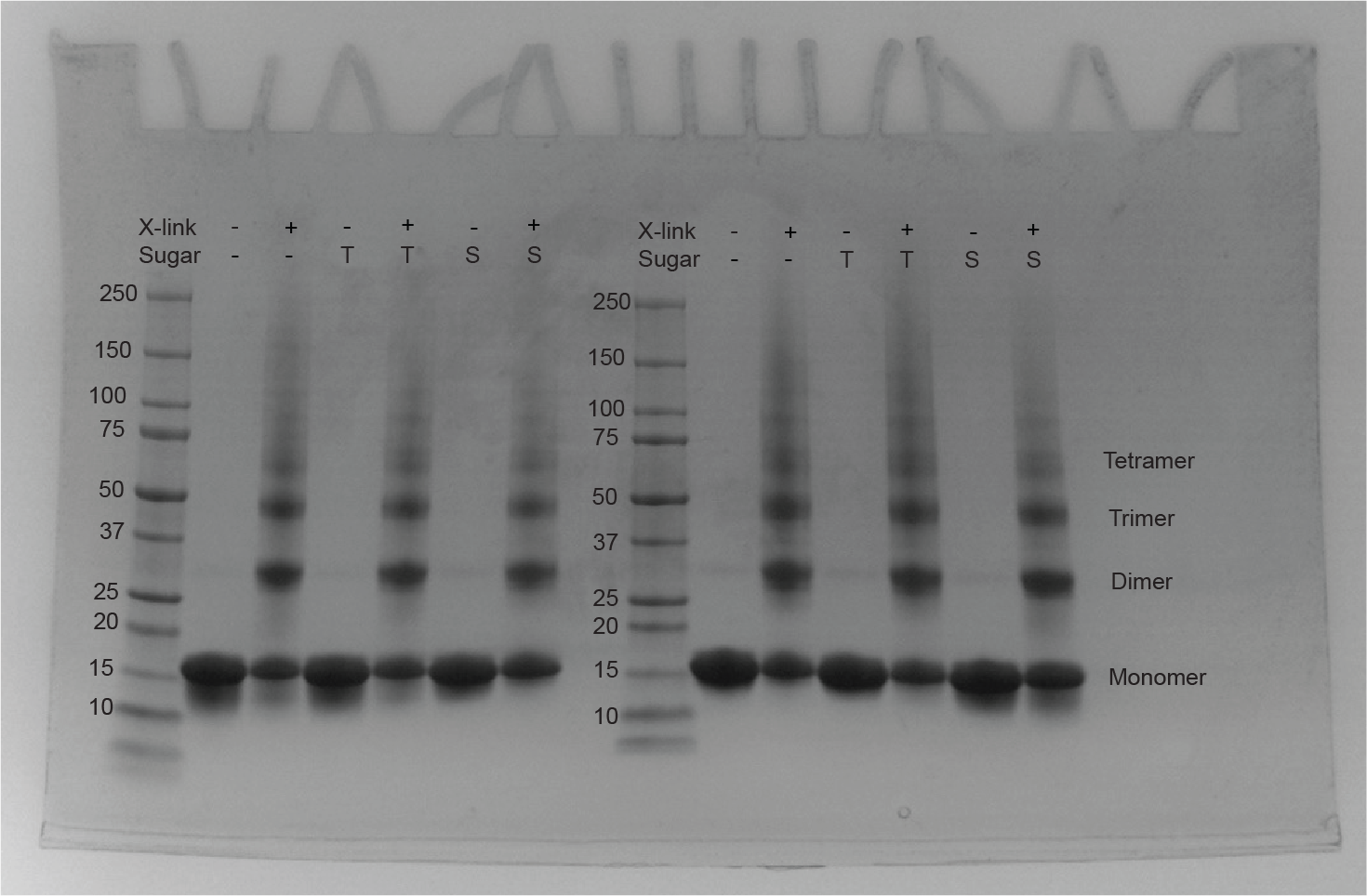


**AtLEA4-2**

1. 25 µM B) 50 µM

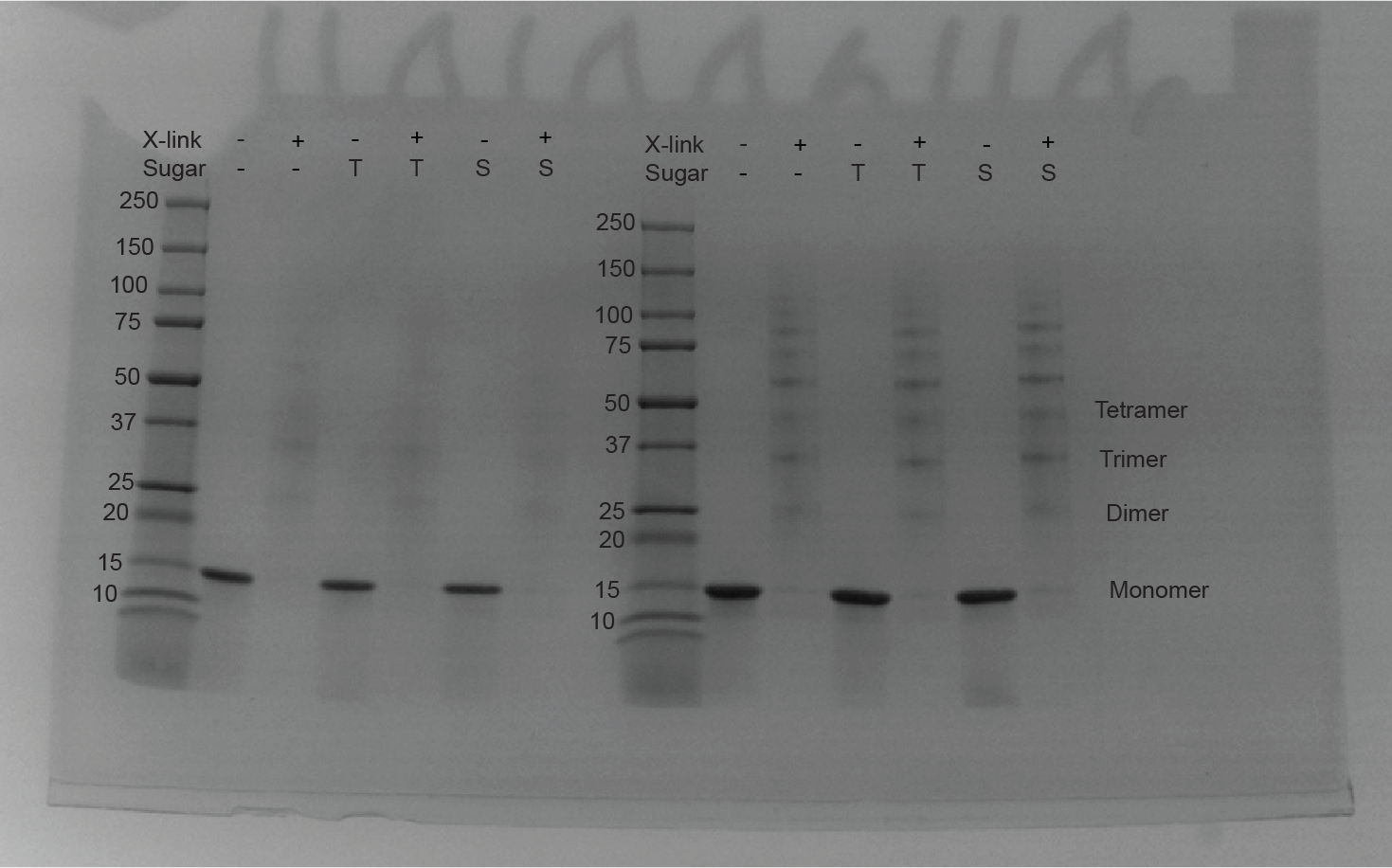


C) 75 µM D) 100 µM

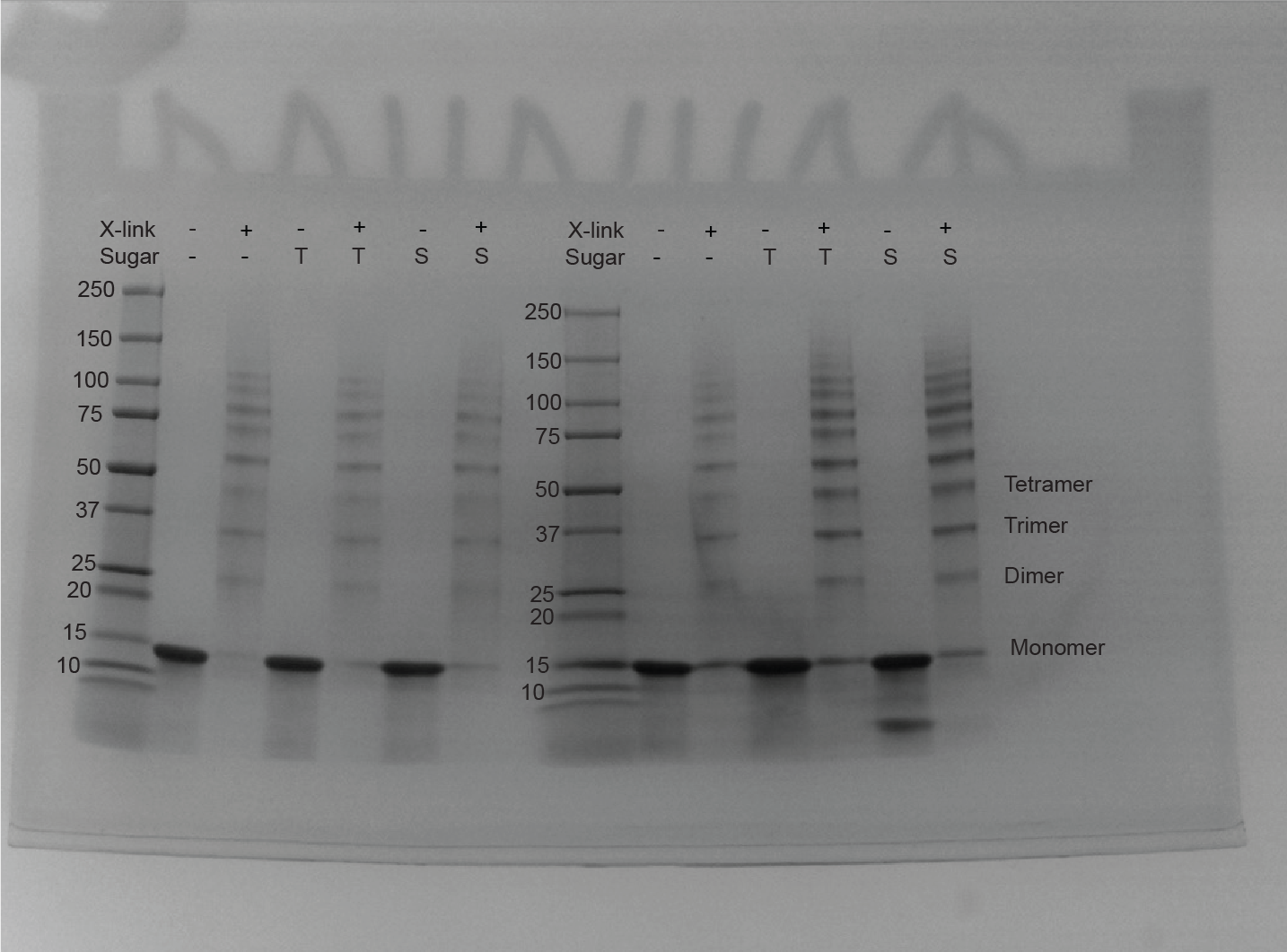


E) 150 µM F) 200 µM

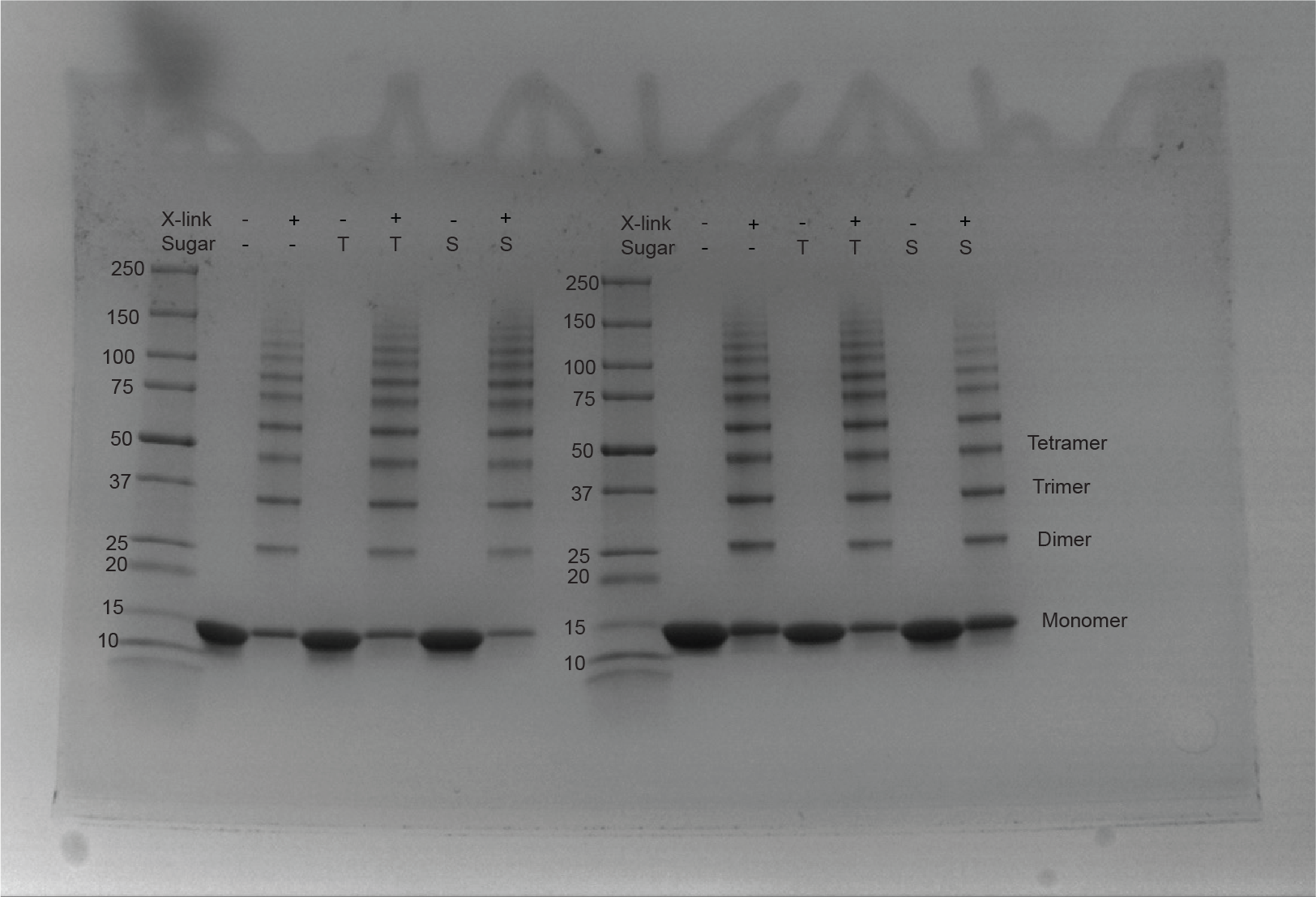


**AtLEA3-3**

A) 25 µM B) 50 µM

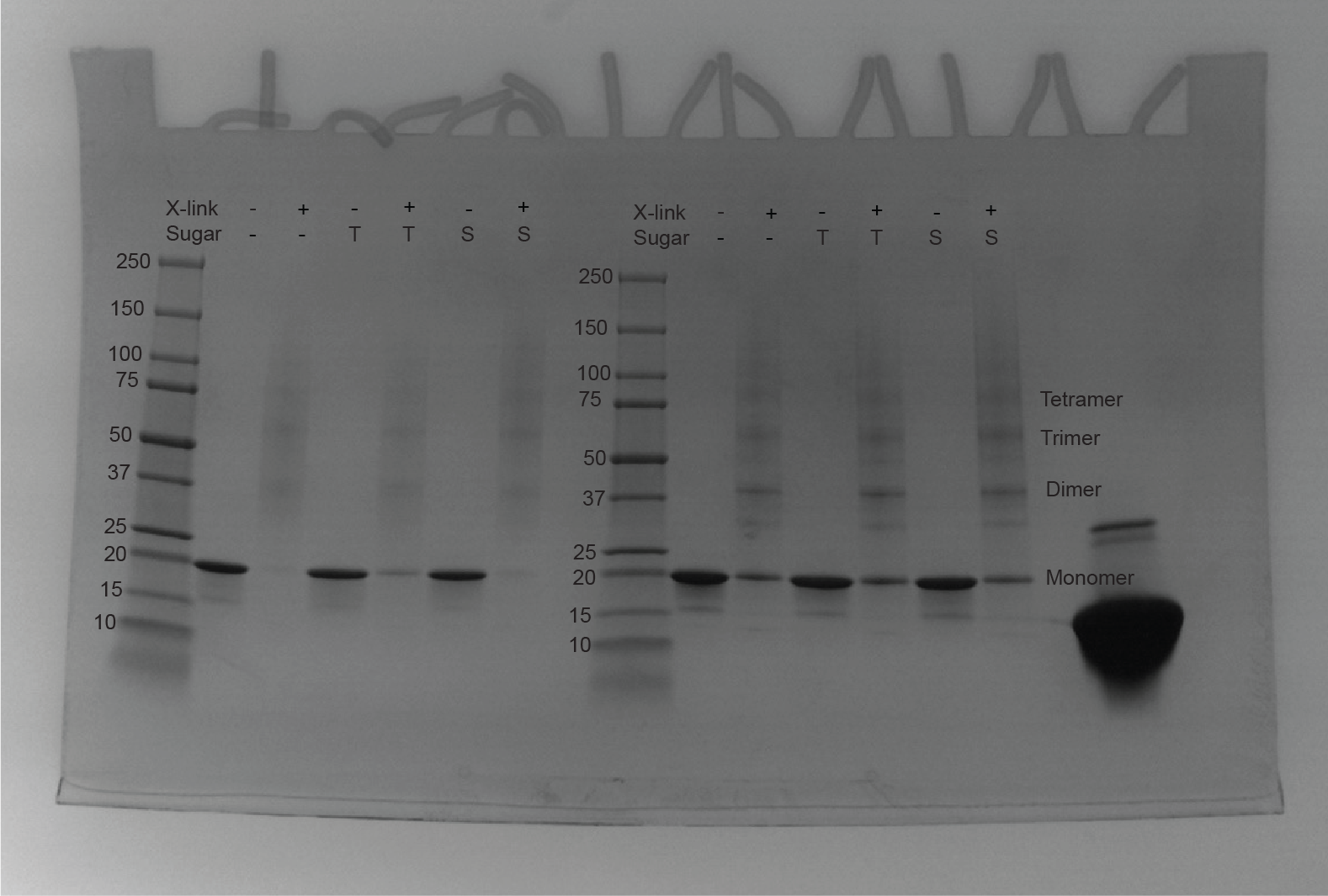


C) 75 µM D) 100 µM

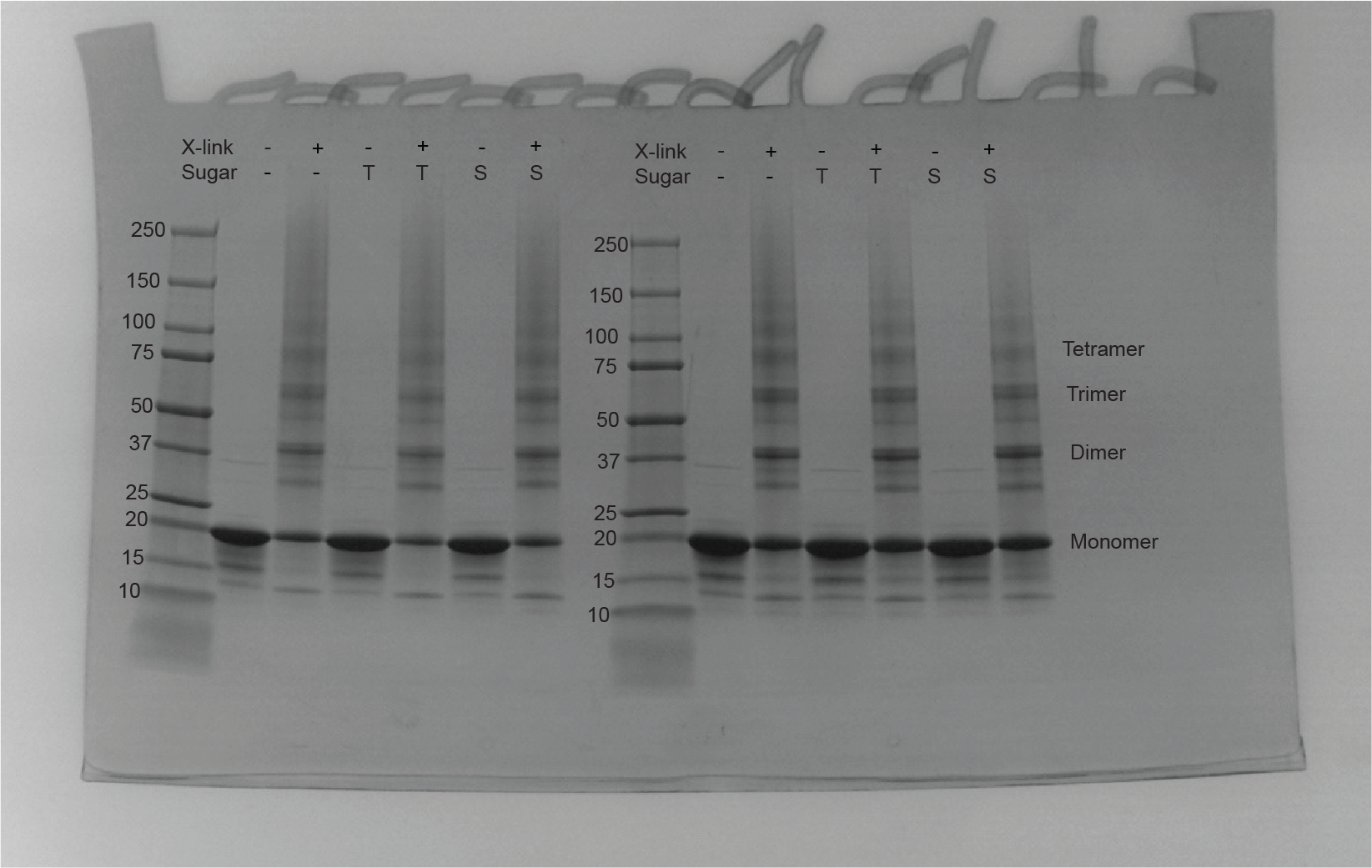


E) 150 µM F) 200 µM

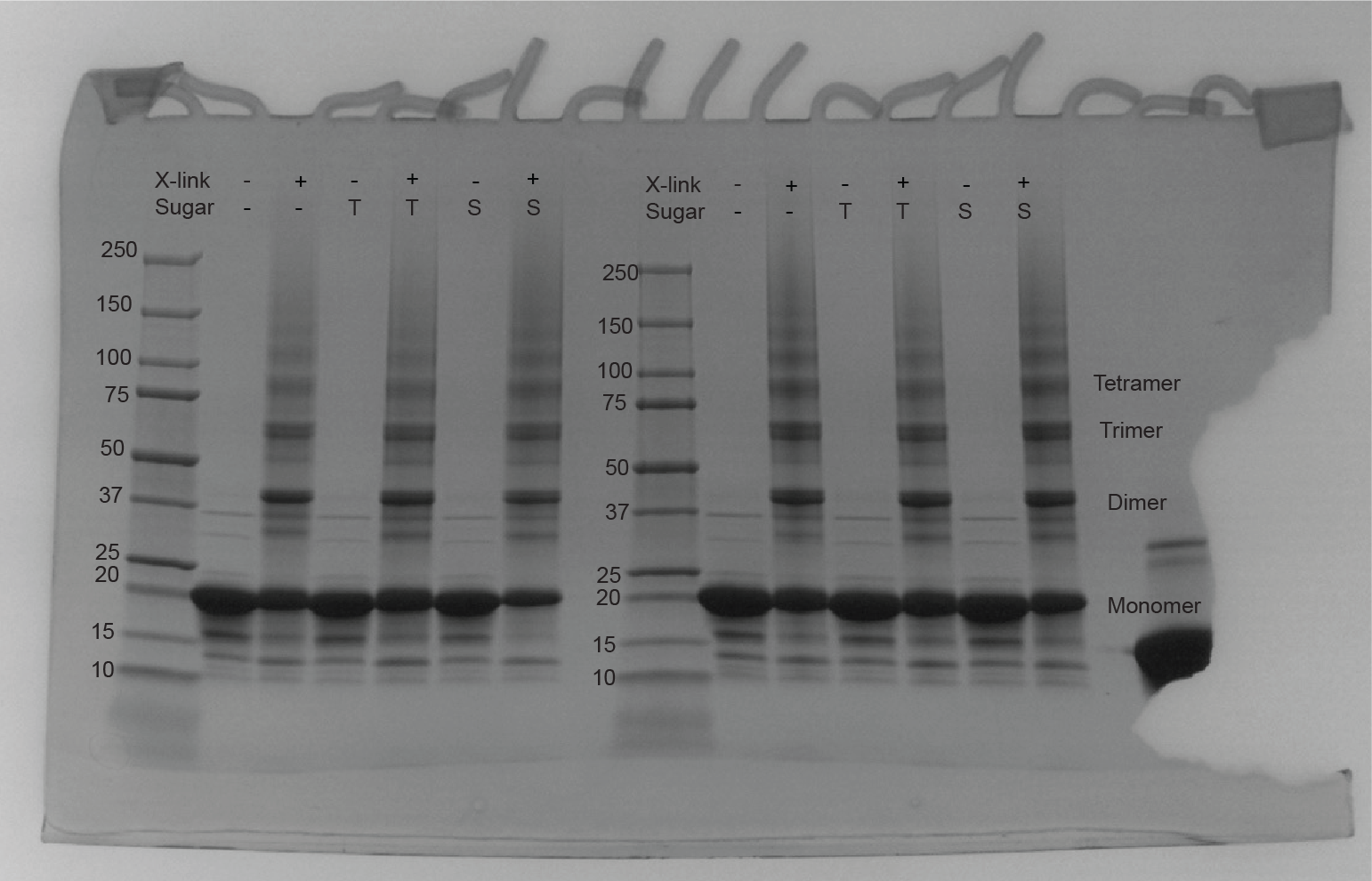


**HeLEA68614**

A) 25 µM B) 50 µM

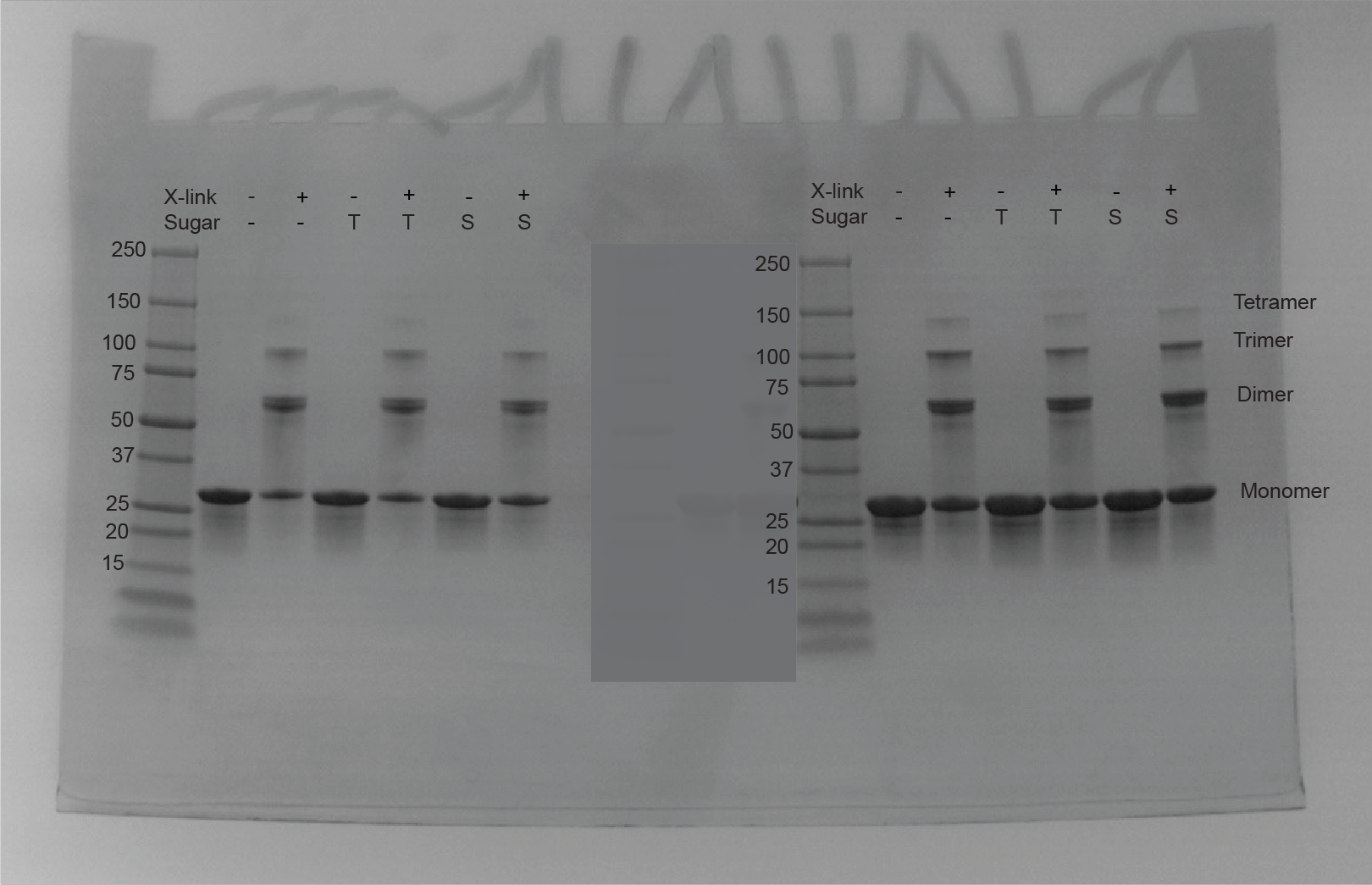


C) 75 µM D) 100 µM

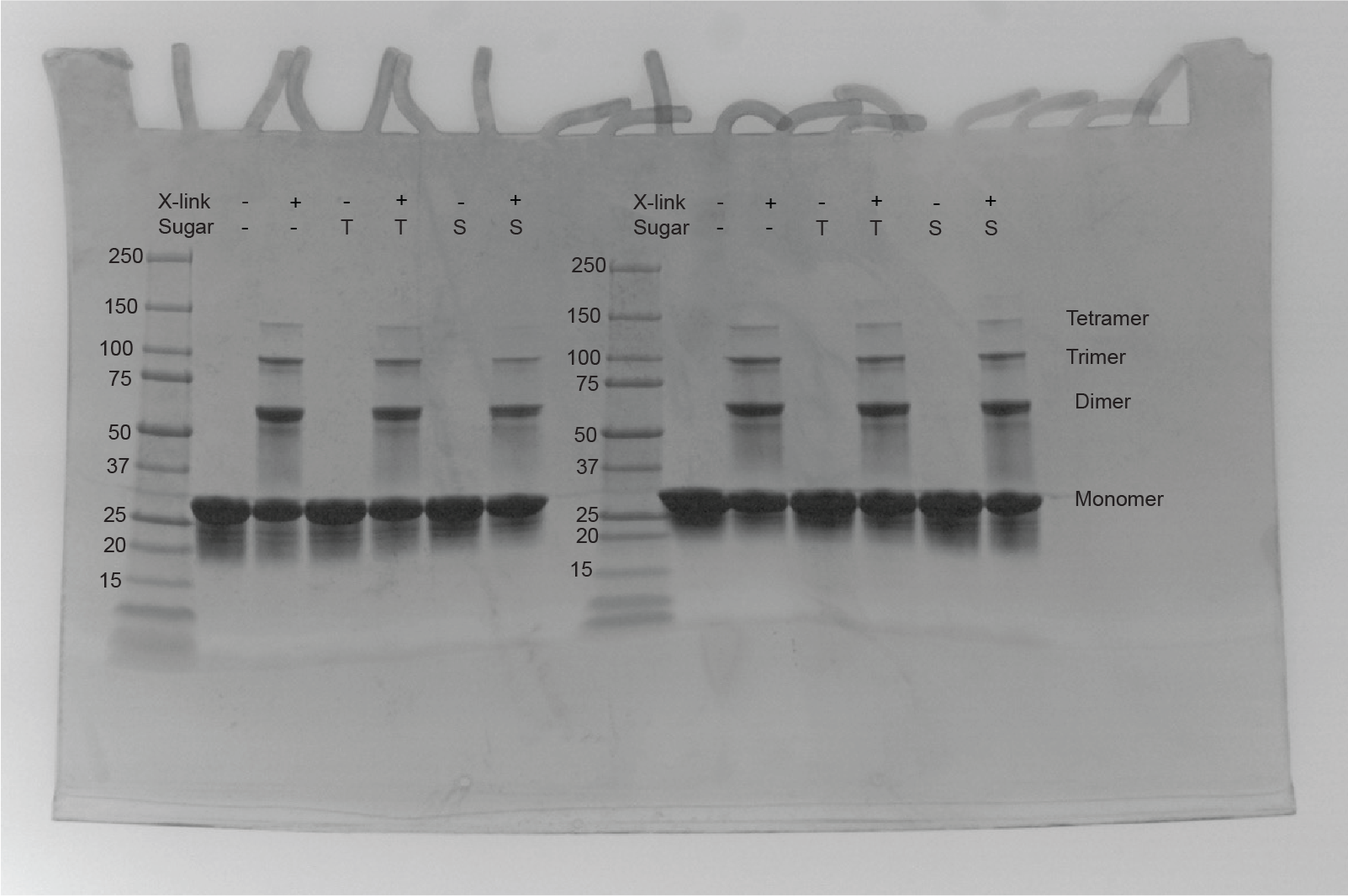


E) 150 µM F) 200 µM

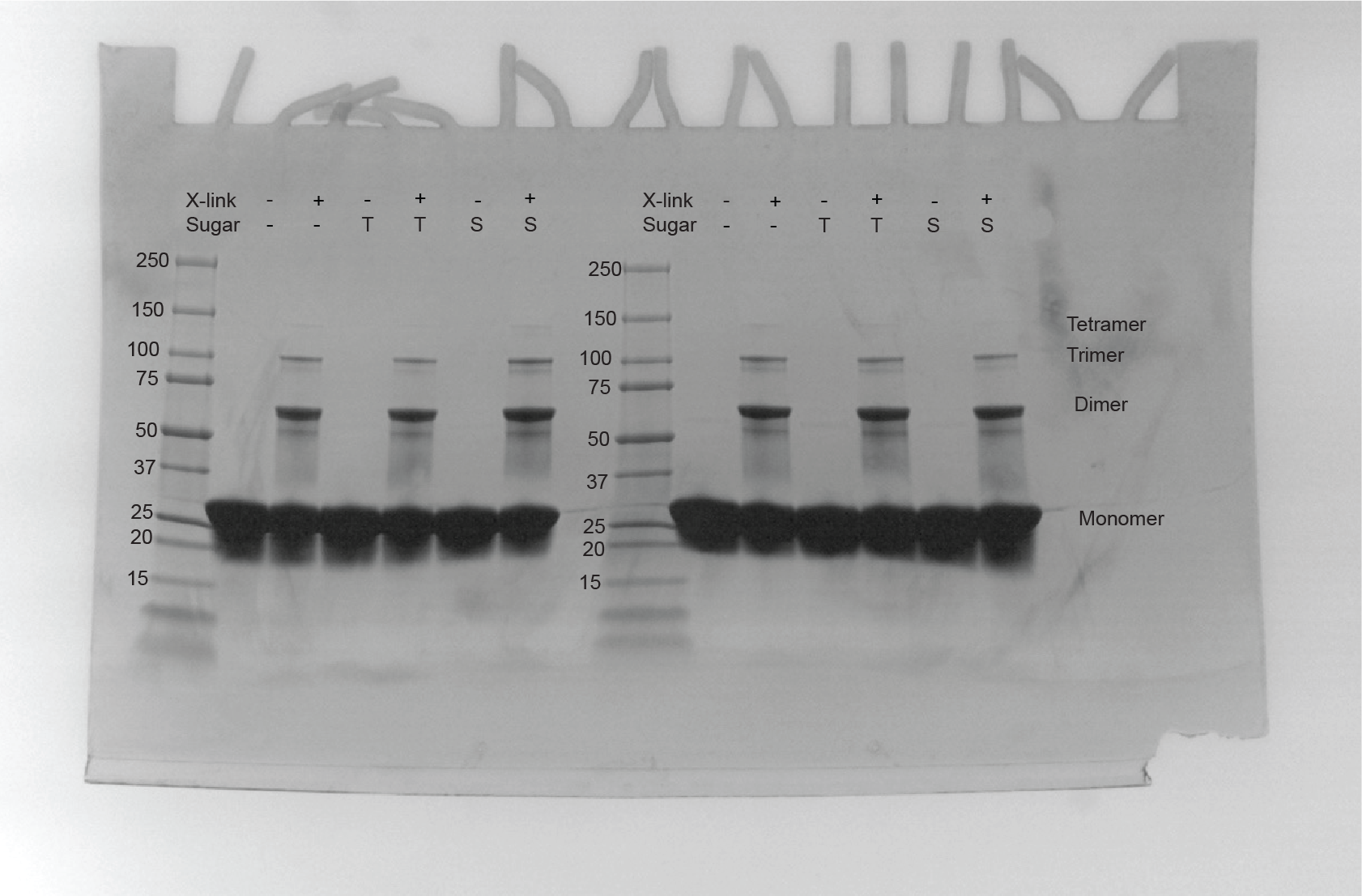


**AvLEA1C**

A) 25 µM B) 50 µM

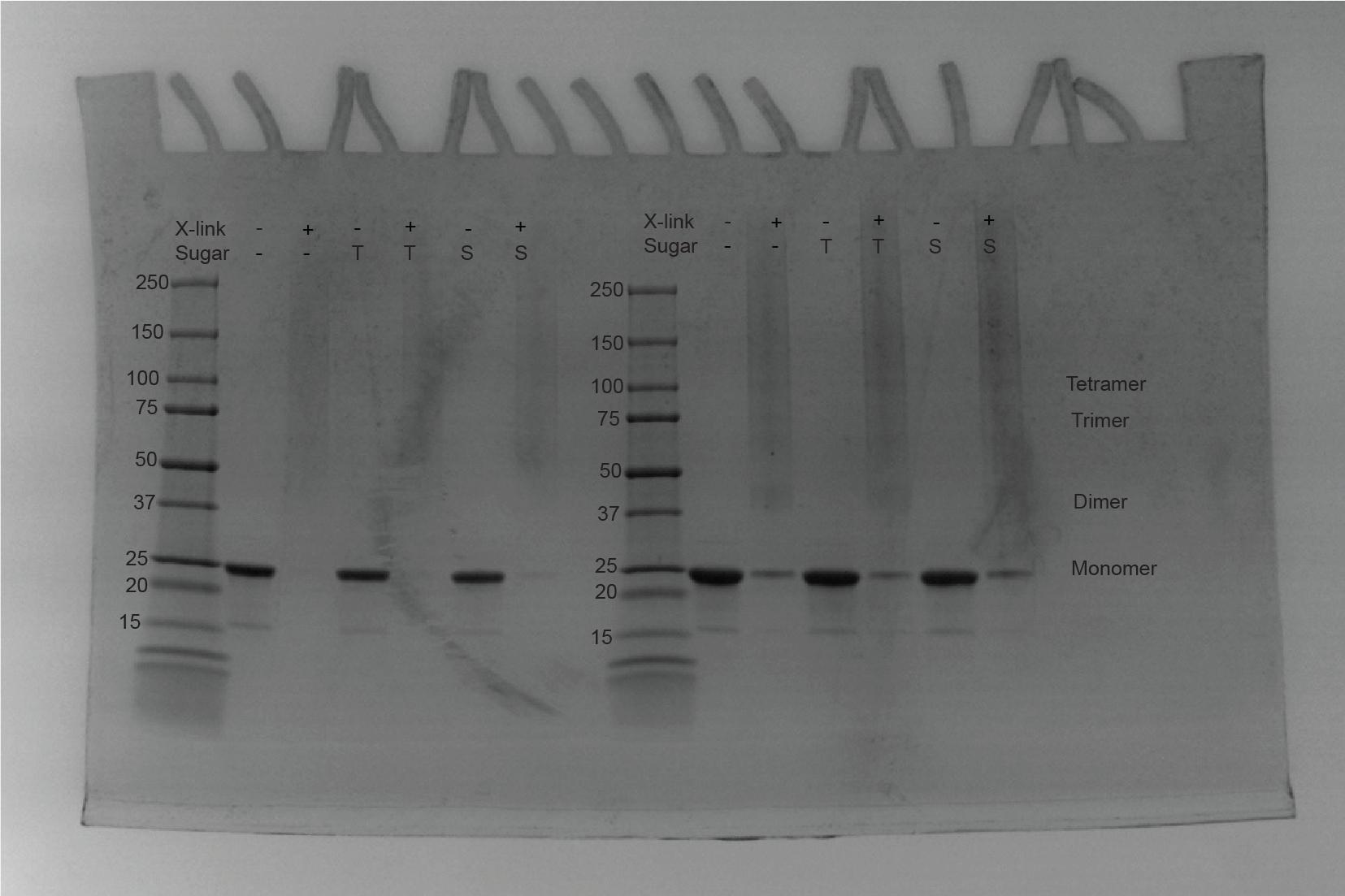


C) 75 µM D) 100 µM

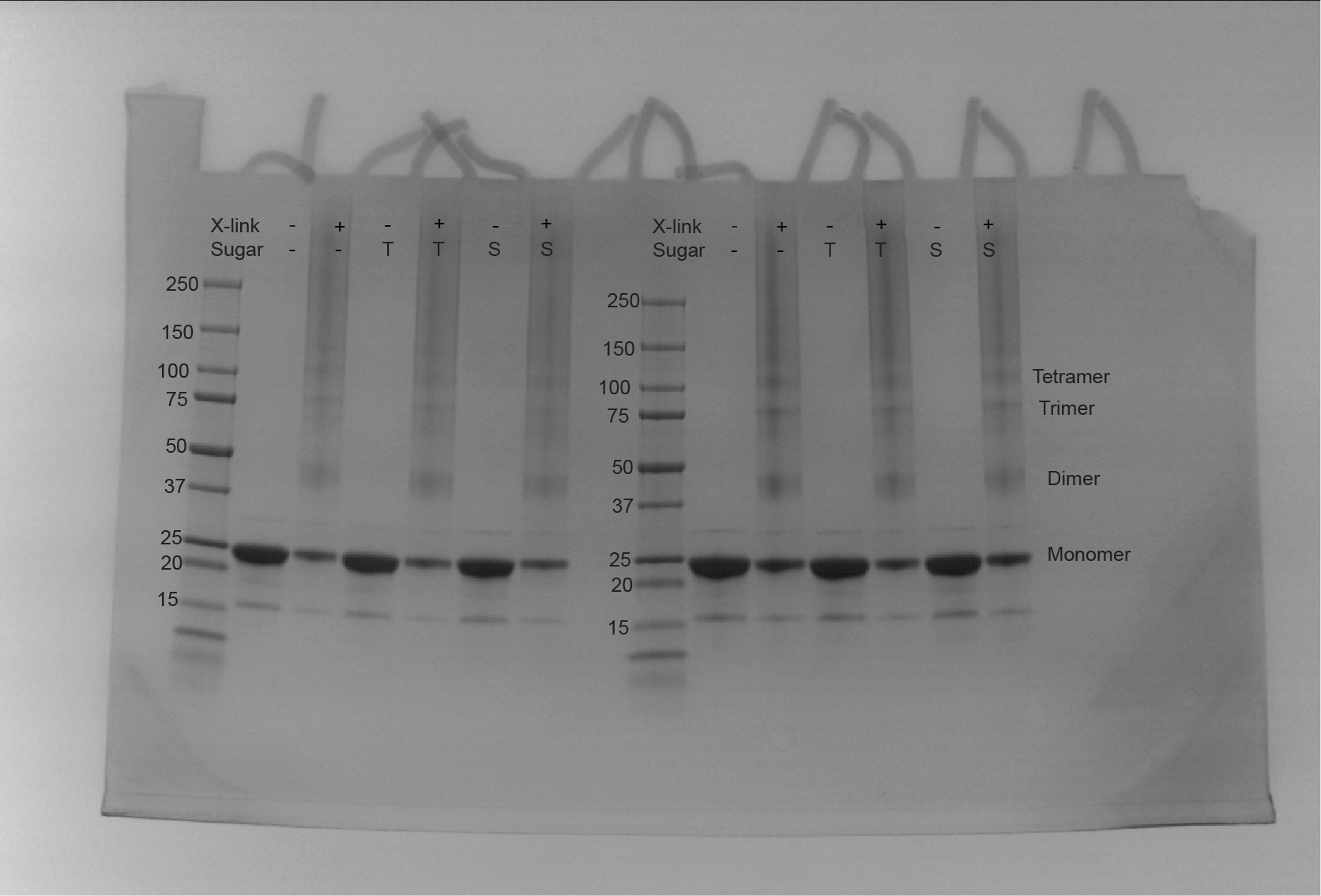


E) 150 µM F) 200 µM

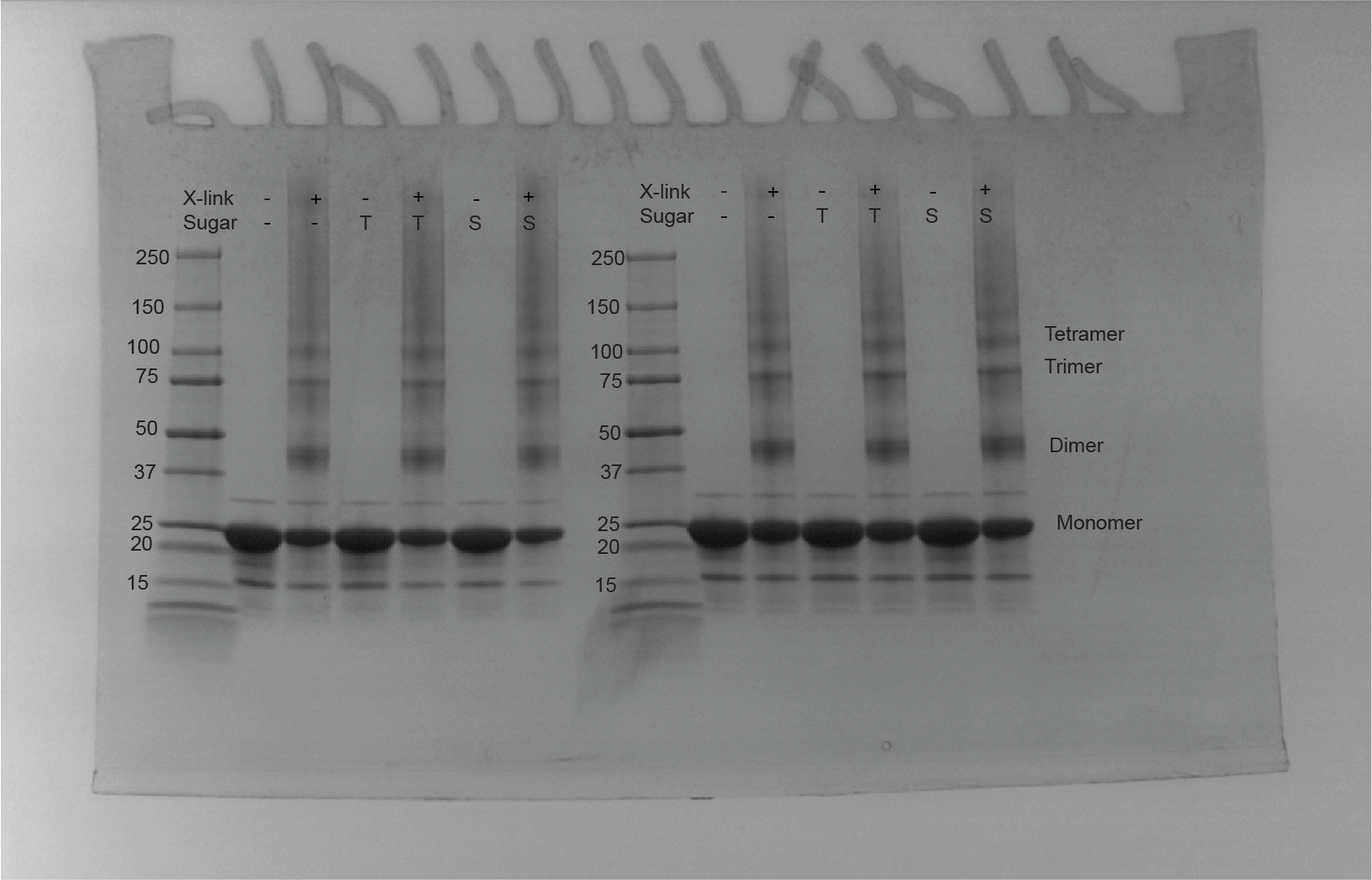

Supplement: Figure 4—figure supplement 2—source data 2. [file elife-97231-fig4-figsupp2-data2.docx]
