# Supplementary material for: Disordered proteins interact with the chemical environment to tune their protective function during drying
Source: eLife. 2024 Nov 19;13:RP97231. doi: 10.7554/eLife.97231 (PMC11575898; doi:10.7554/eLife.97231)
Supplement: Supplementary file 1. [file elife-97231-supp1.pdf]

Organism	Motif	Concentration (mM)
	At11	0.297
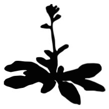	At22	0.277
	At44	0.161
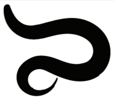	Aav11	0.0562
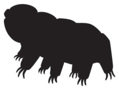	He11	0.287
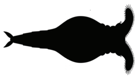	Av11	0.262
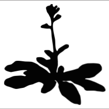	At20	0.264

A. Protein concentrations used in synergy assays for LEA motifs in this study.

Organism	Protein	Concentration (mM)
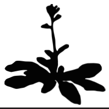	AtLEA3-3	0.1215
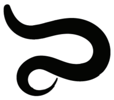	AavLEA1	0.112
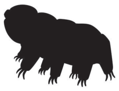	HeLEA68614	0.0392
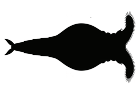	AvLEA1C	0.068
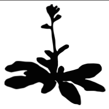	AtLEA4-2	0.217
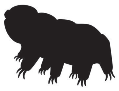	CAHS D	0.0078
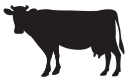	BSA	0.054

B. Protein concentrations used in synergy assays for full-length proteins in this study
