# Supplementary material for: Disordered proteins interact with the chemical environment to tune their protective function during drying
Source: eLife. 2024 Nov 19;13:RP97231. doi: 10.7554/eLife.97231 (PMC11575898; doi:10.7554/eLife.97231)
Supplement: Supplementary file 2. [file elife-97231-supp2.doc]

**LEA motif sequences**

**>At11**

AKSKADETLES

**>Aav11**

LRDTAAEKLHQ

**>He11**

LKDKAGSAWNQ

**>Av11**

KDKAAEALDAI

**>At22**

AKSKADETLESAKSKADETLES

**>At44**

AKSKADETLESAKSKADETLESAKSKADETLESAKSKADETLES

**>At20**

AKEKLNIGGAKAQGHAEKTM

**Full-length proteins**

**>AtLEA3-3**

MASHQEQSYKAGETRGKAQEKTGEAMGTMGDKTQAAKDKTQETAQSAQQKAHETAQSAKDKTSQAAQTTQERAQESKDKTGSYMSETGEAIKNKAHDAAEYTKETAEAGKEKTSGILGQTGEQVKQMAMGATDAVKHTLGLRTDEGNKEHVSSAPSTTTTTTTRETQRK

**>AavLEA1**

MSSQQNQNRQGEQQEQGYMEAAKEKVVNAWESTKETLSSTAQAAAEKTAEFRDSAGETIRDLTGQAQEKGQEFKERAGEKAEETKQRAGEKMDETKQRAGEMRENAGQKMEEYKQQGKGKAEELRDTAAEKLHQAGEKVKGRD

**>HeLEA68614**

MFLARNVSRVALRSVSLSPAAIPQQQHAGVAAVYAVRFASSSGSGRPADNWAESQKEKAKAGLKDAQAEVGKVAREVKDKAAGGIEQAKDAVKQGANDLKRSGSRTFENAKDDIQAKAQHAKSDLKGAKHQAEGVVENVKEAAENAWEKTKDVAENLKDKVQSPGGLADKAANAWETVKDRAQDAASEVKHKAGDLKDKAQQVIHDATTQSGDNRKQDQQQRRDSQGSQSGQNSRSRN

**>AvLEA1C**

MNKFLSILCLVLCISATFAKQSATEQAVNAAADLKDKVKDAASAAYDAASPKVAEGAEFIKDKAEQAYETGSKVAGEYADVAKEKLAKVADDVKASAQNFANDASKTGQEYAQEGLKQGQKLGEQAFEVGKDKANEALKAAQKSGADAYEAALEYGADGVKRAKQLPEQTVELSRDKANEALKAARHAAGDSIDSATEYVQETRKQASKKAKETTEEASEKAQKAKRNADL

**>AtLEA4-2**

MQSAKEKISDMASTAKEKLNIGGAKAQGHAEKTMARTKKEKKLAQEREKSKEAQAKADLHQSKAEHAADAQVHGHHLPGHSTYPTRATGANYPPGQI

**>CAHS D**

MSGRNVESHMERNEKVVVNNSGHADVKKQQQQVEHTEFTHTEVKAPLIHPAPPIISTGAAGLAEEIVGQGFTASAARISGGTAEVHLQPSAAMTEEARRDQERYRQEQESIAKQQEREMEKKTEAYRKTAEAEAEKIRKELEKQHARDVEFRKDLIESTIDRQKREVDLEAKMAKRELDREGQLAKEALERSRLATNVEVNFDSAAGHTVSGGTTVSTSDKMEIKRN
